# Supplementary material for: Research and analysis of an enhanced genetic algorithm identification method based on the LuGre model
Source: PLoS One. 2025 Jun 3;20(6):e0322844. doi: 10.1371/journal.pone.0322844 (PMC12133191; doi:10.1371/journal.pone.0322844)
Supplement: Attachment — Submitted filename: Review.pdf [file pone.0322844.s002.pdf]

Research and Analysis of an Enhanced Genetic Algorithm Identification Method Based on the LuGre Model

Comments to improve:

١. In abstract and also in the introduction there were some phrases that was repeated similarly, it's better to have a different type of explanation and more summarized in abstract.
٢. It is better to also have an explanation on how to calculate the target friction force based on motor dynamic equations.
٣. The arrows in Fig.٢ could have been shown more understandable.

Strength Points:

١. Having a comparation between suggested controller and another type to display the improvement and efficiency.
٢. Mentioning the strength and week points of each method used, in introduction.
٣. Good breakdown of parameters to identify.
٤. Defining the LuGre equations in full term and not simplifying it.

Week Points:

١. In introduction it is better to focus on evaluated of LuGre model instead of mentioning the history of this method.

٢. The equations of LuGre coefficients in Eq.٢ could have been introduced separately.

٣. At section "Improved Annealing GA" the dictation of LuGre is incomplete.

٤. The input of flowchart is incorrect at Fig.٣.
٥. The error between two plots is considerable at Fig.١١.

Questions:

١. What was the case of choosing a liner motor in order to evaluate a nonlinear friction model?
٢. In Eq.١ the full formula has a parameter α as the power of e, why in this equation it was determined ٢ and no other values?
٣. On which contact surface do we have the deflection of bristles?
٤. Why the identification matrix does not contain parameter $\sigma_{۲}$?
٥. In Eq.٦ the parameter "k" stands for number of irritations or time step since the parameter $z(t)$ is a time dependent variable?

The up to date references should be added to survey.

[١] P. Moradi, M. H. Korayem, N. Yousefi, "Extended Nonlinear Time-varying **Lugre**-based Friction Model Identification of Robot Manipulator with SMC Compensation Approach", Proceedings of the Institution of Mechanical Engineers, Part I: Journal of Systems and Control Engineering ٢٣٧, no. ٢: ٢٠٧-٢١٩, ٢٠٢٣.

[٢] P. Moradi, M. H. Korayem, N. Yousefi, "Online Identification and Robust Compensation of Extended Nonlinear Time-varying **Friction** Model in Robotic Arms", Journal of Mechanical Science and Technology ٣٧, no. ١: ٣٦٧-٣٧٣, Jan ٢٠٢٣.

[٣] M. H. Korayem, M. Zakeri, and M. Taheri, "Simulation of Two-Dimensional Nanomanipulation of Particles Based on the HK and **LuGre** Friction Models", Arabian Journal for Science and Eng., Vol. ٣٨, No. ٦, pp. ١٥٧٣-١٥٨٥, February ٢٠١٣.

[٤] M. H. Korayem, A. Hedayat, and S. F. Dehkordi, "Application of **frictional** contact to extend the functionality of cooperative manipulator chains in moving a common object in the form of closed kinematic chain", Applied Mathematical Modelling, Vol. ٩٠, Pages ٣٠٢-٣٢٦, Feb. ٢٠٢١.
