# Supplementary material for: Research and analysis of an enhanced genetic algorithm identification method based on the LuGre model
Source: PLoS One. 2025 Jun 3;20(6):e0322844. doi: 10.1371/journal.pone.0322844 (PMC12133191; doi:10.1371/journal.pone.0322844)
Supplement: Attachment — Submitted filename: Response to reviewers comments.pdf [file pone.0322844.s004.pdf]

Reviewer 1

In this paper, an improved genetic algorithm is proposed to identify parameters of the Lugre model, and relevant theoretical derivation is carried out, and the effectiveness of the method is verified through simulation and experiment. However, the theoretical derivation of the paper is confused, the format is different, and the information density of the pictures is low, so the simulated and experimental images cannot fully correspond to the text before and after. Here are some specific suggestions.

1) Formula (1) does not give the definition of all variables;

Answer:

We thank the reviewer for helping improve the quality of the manuscript,

Currently, friction feedforward compensation in servo feed systems predominantly relies on the traditional Stribeck friction model, as illustrated in Fig. 1.

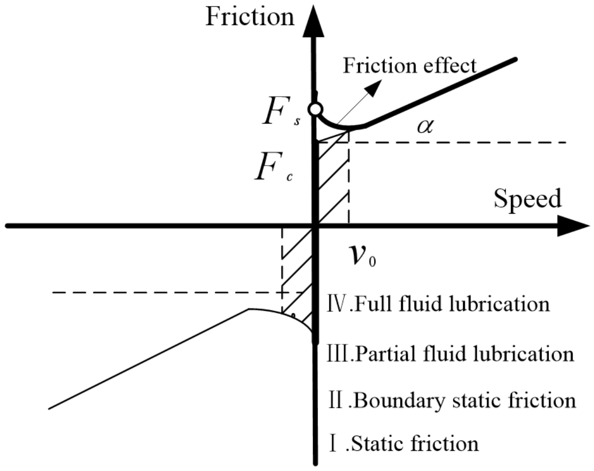

Fig.1 Traditional Stribeck friction model

Traditional friction model expression：

$$F_f(v)=\left[F_c+(F_s-F_c)\cdot e^{-(v\cdot v_s)^{\delta}}\right]\cdot \operatorname{sgn}(v)+\alpha v \tag{1}$$

In Equation (1), $F_f(v)$ represents the total frictional force of the system; v represents the current motion speed of the system, $\mathrm{m/s}$; v_s denotes the

Stribeck speed, $\mathrm{m/s}$; α is the coefficient of viscous friction, $\mathrm{N}s/m$; δ is the Stribeck shape factor, generally, $\delta=2$; F_c refers to the Coulomb friction force, N ; and F_s is defined as the maximum static friction force, N . Consequently, this defines the static coefficient.

We have added the definition of all variables in the manuscript accordingly.

2) Figure 2 is too sloppy, and it is not combined with the article. It only lists the control block diagram of a certain control method, without using the variables given in this article;

Answer:

We thank the reviewer for pointing out this issue, we have changed Figure 2 and fully described using variables given in the article:

In Equation (7) and (8), $u(k)$ is the input of the system; $F_{fr}(k)$ is the actual observed value of the system output, and $F_{fr}(k)$ represents the LuGre target friction force; $\hat{F}_{fl}\left(\hat{\vartheta},\dot{x}(k)\right)$ denotes the estimated model calculation value, which is the LuGre friction force calculated from the identified parameters during the iterative process; $e(k)$ is the estimation error, $e(k)=F_{fr}(k)-\hat{F}_{fl}\left(\hat{\vartheta},\dot{x}(k)\right)$. The fundamental principles of system identification are illustrated in Figure 2.

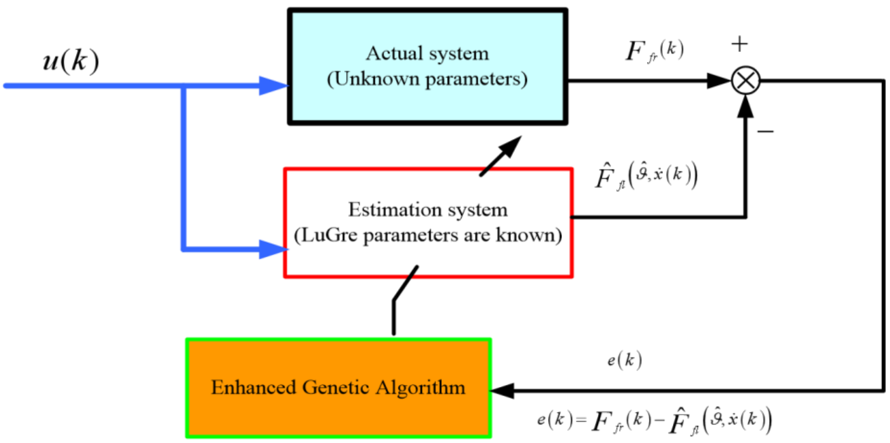

Fig. 2 Basic principles of system identification

3) The mathematical expression of equation (7) is irregular;

Answer:

We are sorry for the confusion, we have adjusted equation 7:

$$e_{\omega}=\int_{t}\omega_{0}(t)-\omega\left(\left[\sigma_{0}\quad\sigma_{1}\quad\sigma_{2}\quad v_{s}\quad F_{s}\right],t\right)^{2}dt \quad (7)$$

4) The words in Figure 3 overlap each other, and it is not clear how to identify parameters, please modify it carefully, and describe the specific process of parameter recognition in combination with the research object and target parameters of this paper;

Answer:

We thank the reviewer for pointing this issue out, we redraw Figure 3 and now it is very clear to identify all parameters, shown as follows:

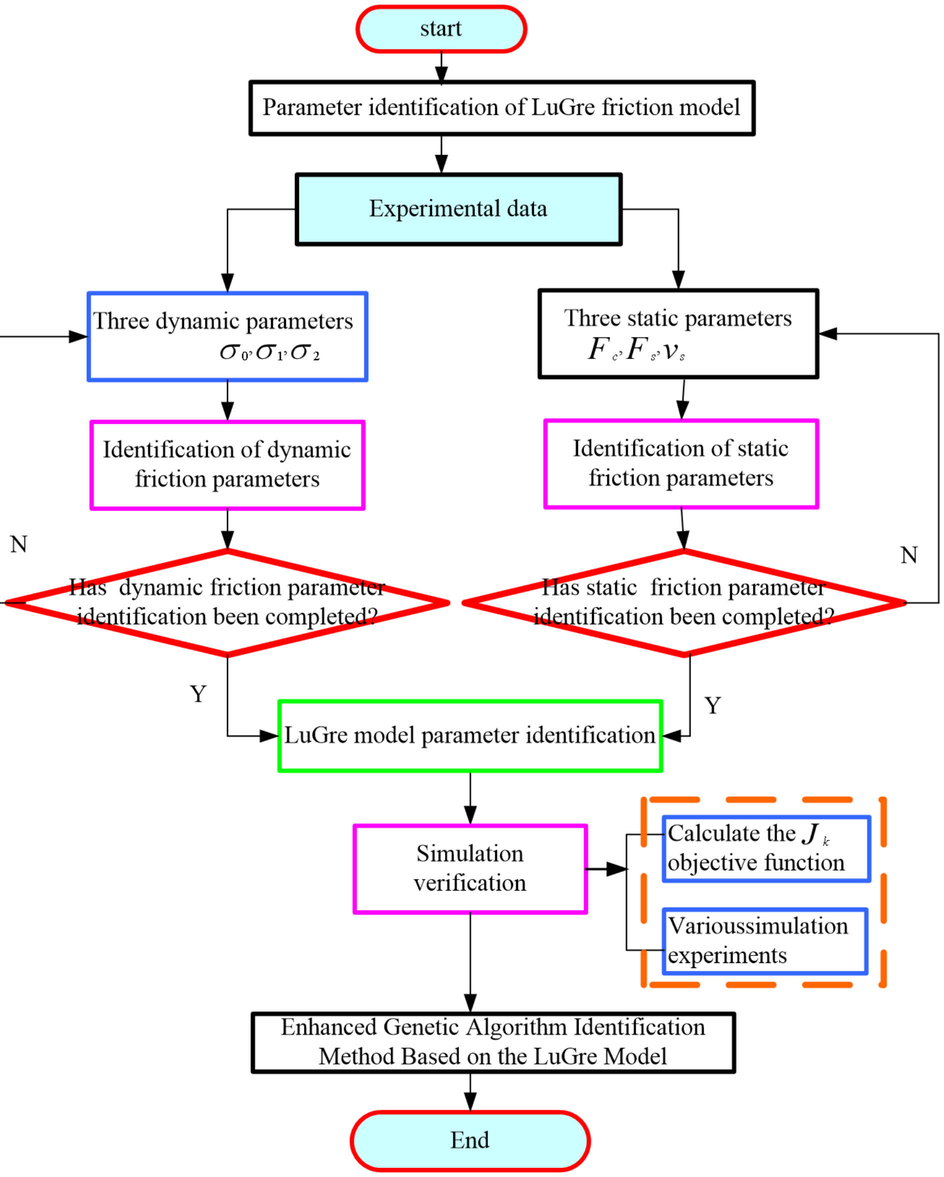

Fig.3 LuGre model parameter identification flow chart

5) Please add experiments to prove the effectiveness of the improved genetic algorithm compared with the traditional algorithm;

Answer:

We thank the reviewer for pointing out this issue, we have added the following contents in the manuscript to reflect the effectiveness of the improved genetic algorithm compared with the traditional algorithm:

The convergence process of the Genetic Algorithm for LuGre parameter identification is shown in Figure 5.

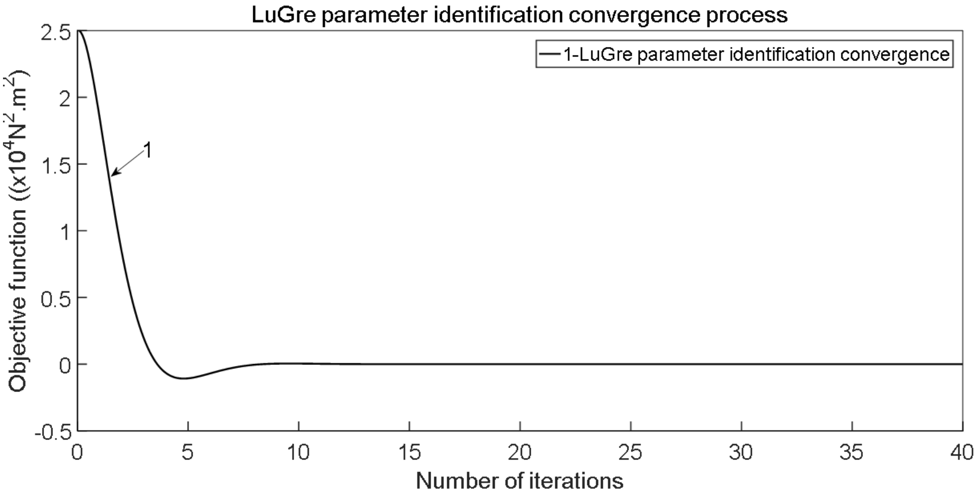

Fig. 5 LuGre parameter identification convergence process

From Figure 5, it can be clearly observed that in the initial stages of the genetic process, the convergence speed is relatively fast. As the number of generations increases, the convergence tends to get trapped in a local optimum, slowing down the speed. When the number of generations reaches a certain level, the Genetic Algorithm escapes the local optimum and gradually converges to the global optimum. Comparison curve of objective function (J_k) is shown in Fig.6.

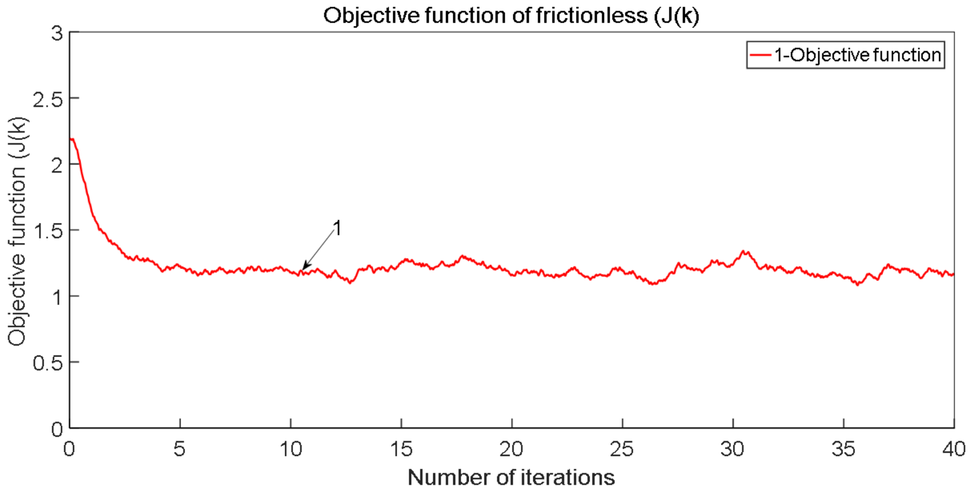

Fig. 6 Comparison curve of objective function (J_k)

The comparative results among the traditional genetic identification algorithm, the adaptive genetic identification algorithm, and the algorithm proposed in this study, along with the corresponding objective function value curves, are illustrated in Figure 7.

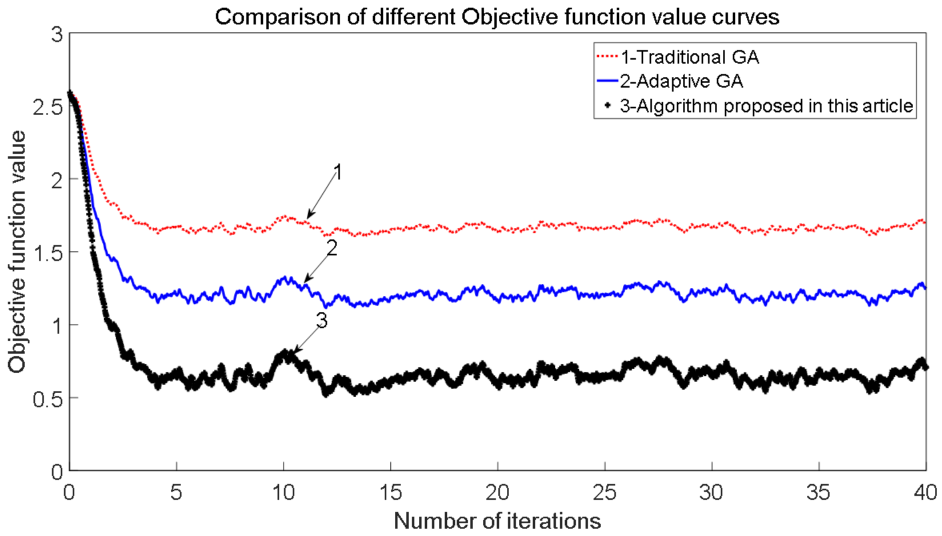

Fig. 7 Comparison of different Objective function value curves

Figures 6 and 7 present a comparison of the traditional genetic identification algorithm, the adaptive genetic identification algorithm, and the algorithm proposed in this paper, along with the corresponding objective function value curves. The results demonstrate that the identified friction force closely aligns with the model, exhibiting minimal error. However, in cases where the identified maximum static friction force significantly exceeds the model's value, this discrepancy may be attributed to testing errors in the provided data. Although some data points fall outside the expected range, they have not been excluded from the analysis. In comparison to the other algorithms, the proposed algorithm in this study achieves faster and more accurate identification of the friction model parameters.

6) Increase the interpretation of experimental results;

Answer:

We deeply appreciate the reviewer for providing constructive comments and helping the readers better understand the manuscript, we have added paragraphs interpreting the experimental results:

To evaluate the influence of bristle stiffness on friction torque, the LuGre model was employed to analyze the tracking accuracy of friction torque and the behavior under micro-displacement and micro-velocity conditions. The

experiment was designed using the Matlab Simulink simulation module and subsequently implemented in TwinCAT3 software. The system was simulated and analyzed with the following parameters: simulation inputs set to $v(t)=4\sin(2\pi t)$, a simulation duration of 3 seconds, a fixed-step simulation type with a step size of 0.0001 seconds, and the solver configured to use the ode4 method. The LuGre parameters were simulated and compared separately using both the original and identified parameters. The corresponding time-dependent friction torque curves and identification results are illustrated in Figure 20, which also includes the speed-friction torque curve.

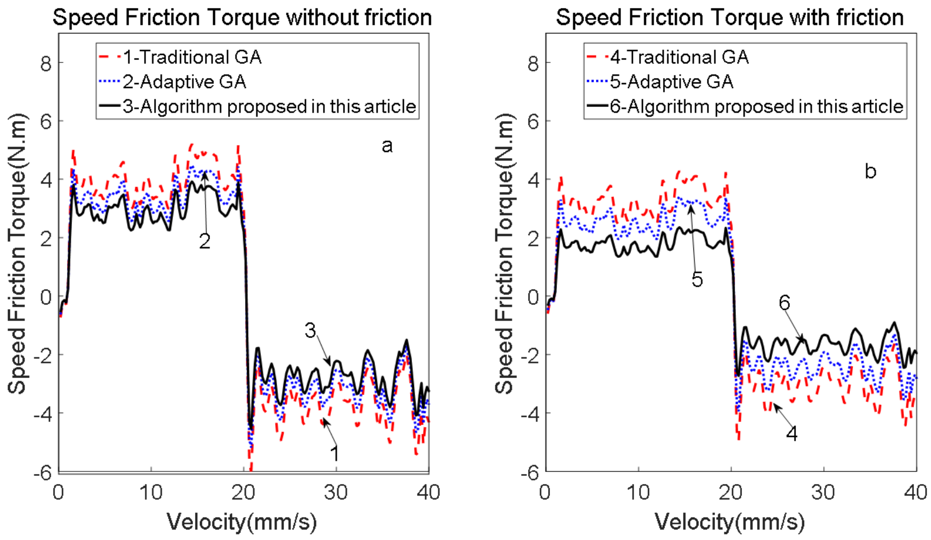

Fig. 20 Comparison results of different speeds and friction torques

From Figure 20, it is evident that when the friction torque crosses the zero point during upward and downward motion, the tracking error waveform becomes distorted and exhibits a peak phenomenon. This indicates that the stiffness of the bristles significantly influences the friction torque.

7) Please carefully check the control methods compared in the experiment, and there are many language expression errors in the text, such as : (1) The upper part of Figure 14 represents the control effect of fuzzy PID, and the lower part represents the control effect of traditional PID ; (2) The lower part of Figure 15 shows that the fuzzy PID control system combined with friction feedforward compensation is proposed in

this paper, which is inconsistent with the theme of this paper.

Answer:

We thank the reviewer for pointing out these errors in Section 5, we have completely remodified the section and the revised contents are as follows:

5.2 Friction Compensation Experiment

(1) Experimental Study on LuGre Model Feedforward Friction Compensation Controller

To mitigate the adverse effects of nonlinear friction on system performance, a feedforward friction compensation controller based on the LuGre model was designed. The control architecture employs a three-loop strategy, where the current loop is embedded within the motor driver, while the speed and position loops are externally implemented. The controller was developed in Simulink and deployed via TwinCAT3 software for real-time execution.

The compensation scheme utilizes feedback speed as the input to the LuGre friction model. To address sampling-period delays inherent in feedback-based compensation, a feedforward friction compensation structure (Fig. 21) was adopted. This structure directly injects the estimated friction force into the control input, bypassing latency issues and enhancing system responsiveness.

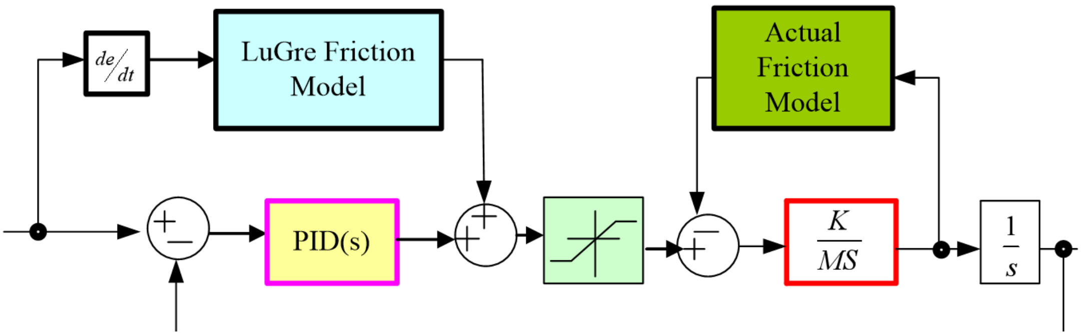

Fig. 21 Feedforward friction compensation model

By introducing a sinusoidal signal $x(t)=15[\cos(\pi t)-\pi/6]$ into both control systems, the signal tracking performance is analyzed. The experiments are divided into two groups based on the presence or absence of feedforward compensation for comparative analysis. The speed tracking curves before and after friction compensation are illustrated in Fig. 22.

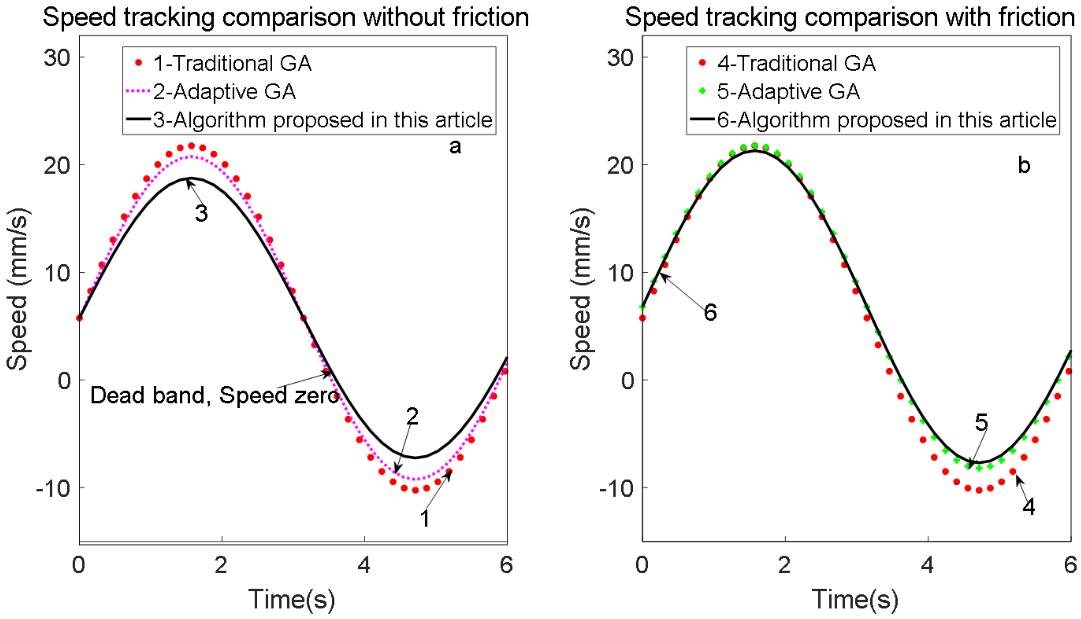

Fig. 22 Speed tracking curve before and after friction compensation

As illustrated in the speed tracking results, a 'dead zone' is observed during zero-speed transitions. The implementation of friction feedforward compensation significantly mitigates this issue, achieving minimal position tracking error and smooth velocity transitions at the zero-crossing point. The position tracking curves before and after the application of friction compensation are illustrated in Fig. 23.

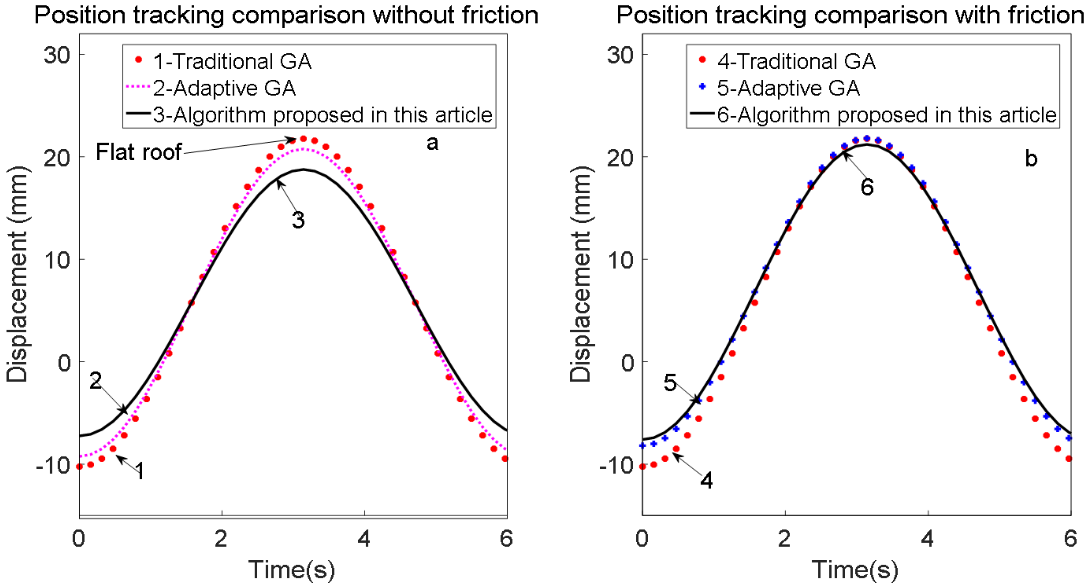

Fig. 23 Position tracking curve before and after friction compensation

The experimental results demonstrate that feedforward friction compensation

control based on the LuGre model significantly outperforms traditional PID control in addressing speed tracking imbalances caused by nonlinear friction. This improvement enhances both speed and position tracking accuracy, showcasing greater practical applicability in engineering contexts.

(2) Control Shear Force and Control Force of Identification Values

For the LuGre model, static parameter identification follows conventional methods, while dynamic parameter identification utilizes sensor outputs (displacement or acceleration) and the control shear force as direct inputs. During identification, the control shear force serves as the target approximation value, simplifying the identification of two dynamic parameters.

For dynamic parameter identification, the servo system's displacement (or acceleration) and control shear force outputs are directly used, with the control shear force acting as the target approximation value. Assuming the dynamic parameter $x_d = \omega_2 = [\hat{\sigma}_0 \quad \hat{\sigma}_1]$ requires identification, the identification error is defined as:

$$e(x_d, t_i) = u(t_i) - u(x_d, t_i) \tag{38}$$

Where, $u(t_i)$ controls the shear force for the servo system, while $u(x_d, t_i)$ model parameter system identifies the output control force.

$$u(x_d, t_i) = F_f(t) + m\frac{d^2 x_i}{dt_i^2} \tag{39}$$

Substituting $z(t) \to z, \dot{z} \to \frac{dz(t)}{d(t)}, \dot{x} \to x$ yields equation:

$$F_f(t) = \hat{\sigma}_0 \cdot z + \hat{\sigma}_1 \cdot \dot{z} + \hat{\sigma}_2 \cdot \dot{x} \tag{40}$$

$$\dot{z} = \dot{x} - \frac{\hat{\sigma}_0}{g(\dot{x})} z \tag{41}$$

The objective function is defined as:

$$J = \frac{1}{2}\sum_{i=1}^{N} e^2(y_d, t_i) \tag{42}$$

During the identification of power parameters, the servo system outputs

$x(t)=0.01\sin(2.2\pi\cdot t)$, and the corresponding identification results for the control force u and friction force are illustrated in Fig. 24. Fig. 25 displays the hysteresis loops of control shear force and control force displacement for the identified values. Throughout the identification process, the parameter population search space is defined as $F_c\in[0,100]$, $F_s\in[0,100]$, $v_s\in[0,0.1]$, $\sigma_0\in[0,1000]$, $\sigma_1\in[0,1000]$, $\sigma_2\in[0,50]$.

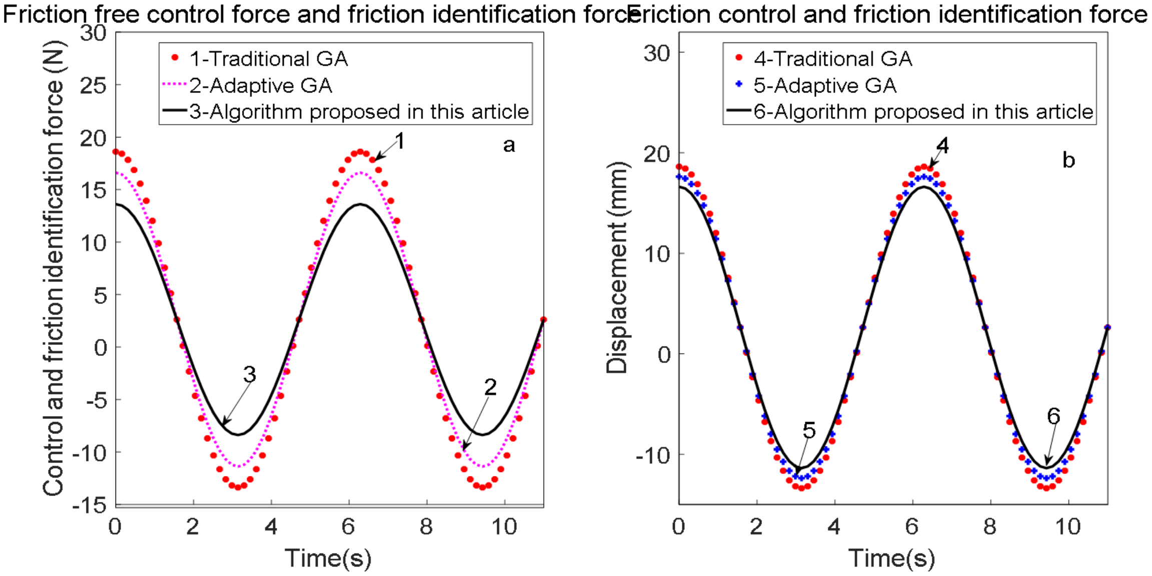

Fig. 24 Control shear force and control force of identification values

It is confirmed that the fuzzy PID control system, integrated with friction feedforward compensation, exhibits both rapid response and steady-state stability. The identification values for friction, control force, and displacement loop are illustrated in Fig. 25.

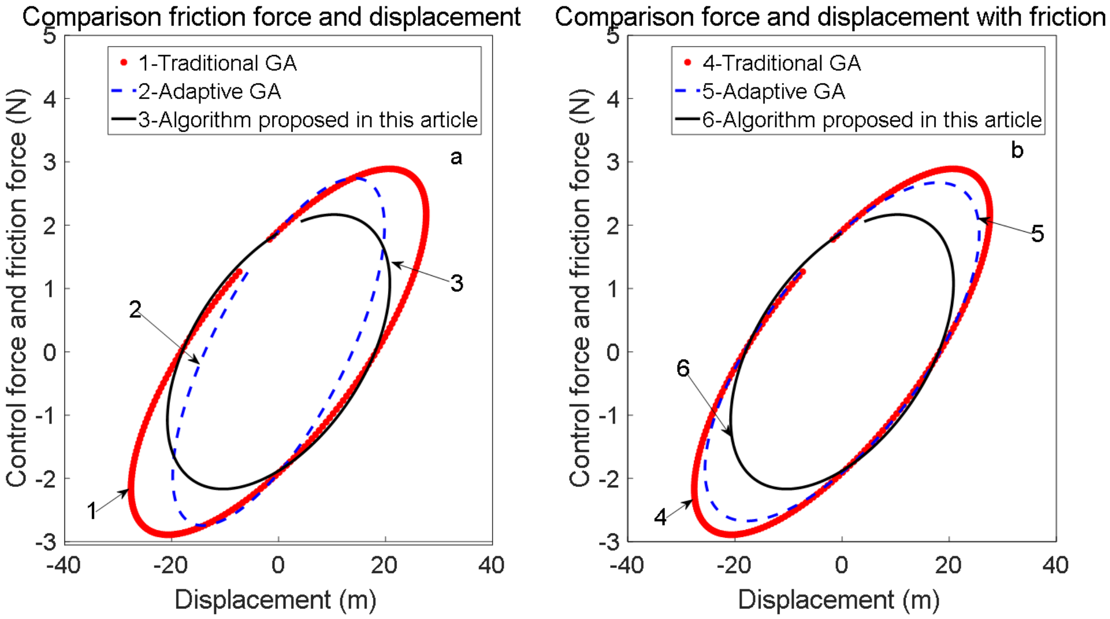

Fig. 25 Friction, control force and displacement loop of identification value

The discrepancy between the friction and control force in the two figures is attributed to inertial force; specifically, the control force is equivalent to the sum of friction and inertial force.

Subsequently, the LuGre friction compensation model was introduced and compared with the simplified model under the same control law, resulting in a significant reduction in tracking error. This fully demonstrates the efficacy of the LuGre friction compensation model.

From the parameter identification results, traditional genetic algorithms (GA), adaptive genetic algorithms, and the proposed algorithm exhibit high accuracy and fast identification speed in determining the static and dynamic nonlinear parameters of the LuGre friction model, establishing them as ideal tools for nonlinear parameter identification.

8) The contribution to method innovation is not significant

Answer:

We thank the reviewer for pointing out this issue, The structure of this paper is organized as follows:

Section 1 introduces the LuGre friction model, detailing its fundamental

principles and mathematical formulation. The LuGre model comprehensively describes both the dynamic and static characteristics of friction in servo systems. Accurate identification of the model parameters is crucial for achieving effective friction compensation.

Section 2 proposes an improved genetic algorithm-based identification method for the LuGre model. This section primarily presents a detailed description of the LuGre friction model and the identification framework.

Section 3 elaborates on the theoretical model of the enhanced genetic algorithm identification method proposed in this paper for the LuGre model.

Section 4 constructs a linear motor platform and outlines its configuration and parameters. It provides a detailed explanation of the parameter identification method for the LuGre friction compensation model and validates the effectiveness of the friction compensation technique as well as the robustness of the designed controller on the physical platform. The performance of the proposed parameter identification algorithm is verified through simulation examples and compared with existing methods.

Section 5 concludes the paper and discusses potential future work.

The main contributions of this paper can be summarized as follows:

1. A novel parameter identification algorithm is proposed by integrating an adaptive evolution module and an optimal solution precision optimization module into the traditional genetic algorithm.
2. Based on simulation experiments, it is demonstrated that the proposed algorithm can effectively identify the six parameters of the LuGre friction model.
3. To highlight the advantages of the proposed method, it is compared with existing approaches. The results show that the method significantly improves the accuracy of parameter identification while ensuring fast convergence. Simulation results validate the effectiveness and feasibility of the algorithm.

Reviewer 2

1) Briefly summarize the improvements made by the proposed friction compensation technique at the end of the abstract. Mention quantitative improvement.

Answer:

We thank the reviewer for pointing out this issue, we have added a sentence that:

This research presents a viable and highly accurate method for identifying parameters in friction models with a maximum improvement of 25.1% in tracking accuracy.

2) Instead of writing 'Reference [5]' , 'Reference [7]', write first author name followed by et al.

Answer:

We thank the reviewer for pointing this out, we have completely rewritten the induction, and first author name is followed by et al., the examples are shown as follows:

"Zhao et al. [16] studied a novel sliding mode control algorithm with adaptive gain, introduced an integral term to design the sliding surface, and selected a torsion control algorithm based on high-order SMC idea as the switching control law."

"Ali K et al. [17] proposed an advanced control algorithm that combines fast integral terminal sliding mode control, robust exact differentiator, and feedforward neural network-based estimator."

"Another work that Ali K et al. [18] proposes is an Integral Sliding Mode Control (ISMC) for an anthropomorphic manipulator using a modified LuGre friction model with friction compensation."

"Furthermore, Ali K et al. [19] proposes a Fault-Tolerant Control (FTC) strategy for a 5-DoF robotic manipulator, combining an observer-based approach with hardware redundancy to enhance performance under actuator and sensor

faults.”

“While Mehmood Y et al. [20] proposes Fractional and Integral Order Fuzzy Sliding Mode Controllers (FSMC, FFSMC) for Skid-Steered Vehicles (SSV) with variable friction coefficients and displaced Center of Gravity (CG).”

“Cong [18] designed an improved Stribeck friction model. Based on the improved model, an inertia stabilization platform and speed stabilization loop feedforward control were designed in the closed-loop control system to avoid vibration and limit cycle problems caused by changes in motion direction and excessive friction compensation.”

“Aguilar Avelar et al. [19] first discussed the modeling, identification, and compensation of the influence of nonlinear friction on flywheel control.”

“Li et al. [20] proposed an improved Stribeck friction model (SFM) and an optimization algorithm consistent with the positioning platform, and verified a compensator based on the friction model and disturbance observer through simulation.”

“Han et al. [21] established a robust positioning control scheme using a friction parameter observer and a recursive fuzzy neural network based on sliding mode control.”

“Meng et al. [22] constructed an adaptive robust controller for dynamic friction compensation based on the LuGre friction compensation model.”

“Lu et al. [24] proposed a sliding mode controller based on the approach law for controlling the position of permanent magnet synchronous motors.”

“Chen et al. [25] proposed a discrete adaptive sliding mode controller for high-precision motion control of linear permanent magnet synchronous motors, which achieves real-time gain tuning capability and robustness, ensuring the performance of tracking tasks. Experimental results show that the designed controller has fast response and strong robustness to uncertainty and noise.”

“Zhao et al. [26] proposed an adaptive neural network nonsingular fast terminal sliding mode control for high-precision position tracking of permanent magnet synchronous motors in the presence of uncertainty.”

3) Also, define all the abbreviations at their first occurrence in the manuscript e.g. PID should be completely defined at its first occurrence.

Answer:

We thank the reviewer for pointing out this issue, in the manuscript we define all abbreviations at their first occurrence include:

Genetic Algorithm(GA);

Proportional-Integral-Derivative Controller (PID)**.**

4) The first paragraph outlining the crucial role of fiction in physical systems could benefit from literature references such as; Adaptive FIT-SMC approach for an anthropomorphic manipulator with robust exact differentiator and neural network-based friction compensation

Answer:

We thank the reviewer for helping improve the quality of the manuscript, we have added a sentence in the manuscript that:

Ali K et al. [17] introduced an advanced control algorithm integrating fast integral terminal sliding mode control (FIT-SMC), a robust exact differentiator, and a feedforward neural network-based estimator. Initially, a dynamic LuGre friction compensation model was considered, and a mechanical manual model for an n-degree-of-freedom system was developed. Subsequently, a nonlinear control method incorporating friction compensation based on FIT-SMC was proposed, demonstrating superior performance compared to traditional compensators.

5) Please fix the overwriting of text and symbols in Figure 3.

Answer:

We thank the reviewer for pointing this issue out, we have fixed the errors and presented a new figure 3 in the manuscript.

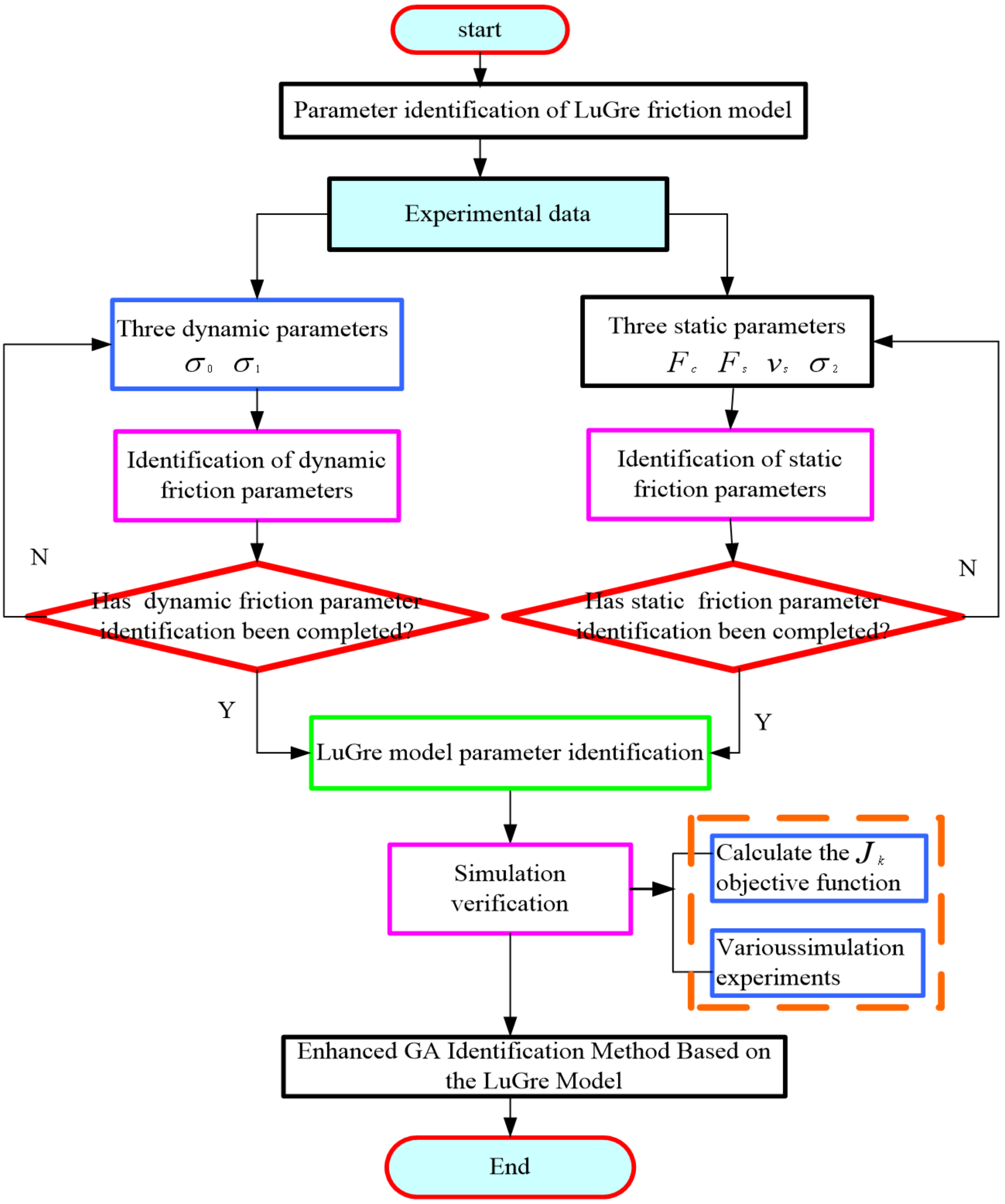

Fig.3 LuGre model parameter identification flow chart.

6) Correct the flowchart in Figure 5. In decision block, are there two different conditions being evaluated? If Yes, is it OR or ND between these conditions? Also, the text of the second last block needs to be updated to "Simulate annealing on the new species".

Answer:

We deeply appreciate the reviewer for being keen on noticing this issue, we have completely redrawn Figure 5 and fixed all issues, the figure is shown as follows:

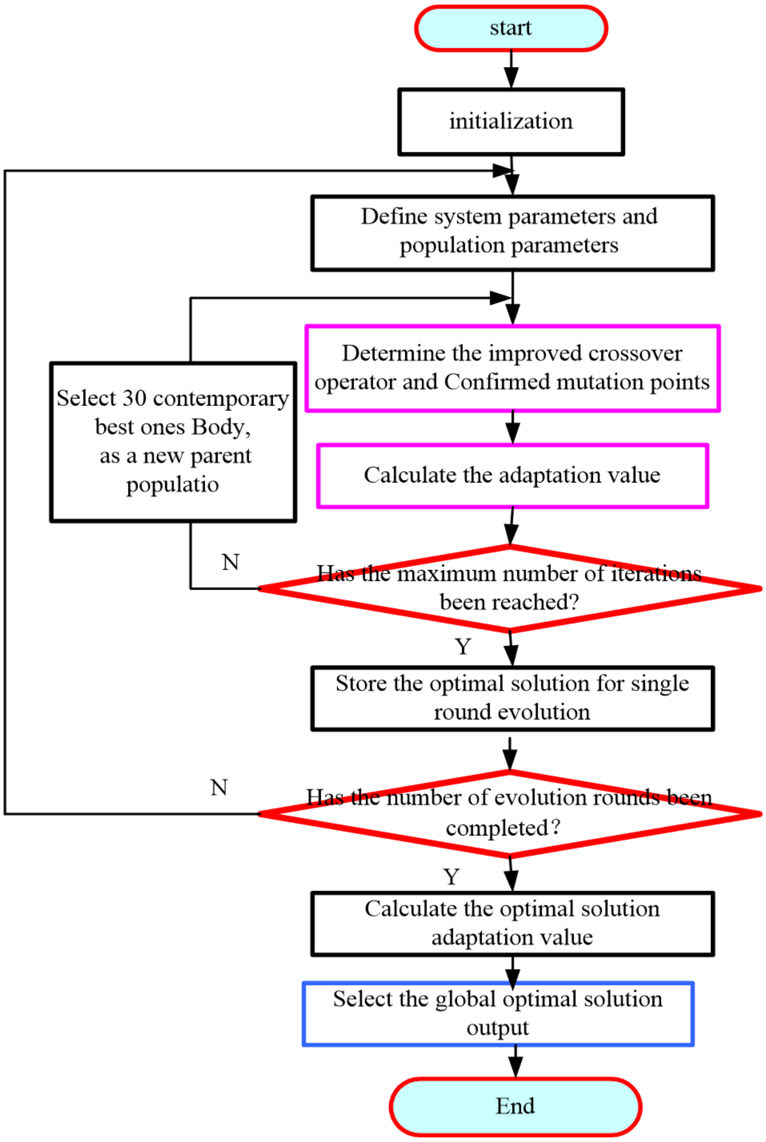

Fig. 5 Flow chart of improved annealing genetic synthesis algorithm

7) Please include paper outlines at the end of Section 1 (Introduction)

Answer:

We thank the reviewer for providing constructive suggestions to the manuscript, we add the following sentences to the manuscript:

The manuscript is composed of five sections. Section 1 introduces the LuGre friction model, detailing its fundamental principles and mathematical formulation. The LuGre model comprehensively describes both the dynamic and static characteristics of friction in servo systems. Accurate identification of the model parameters is crucial for achieving effective friction compensation.

Section 2 proposes an improved genetic algorithm-based identification method for the LuGre model. This section primarily presents a detailed description of the LuGre friction model and the identification framework. Section 3 elaborates on the theoretical model of the enhanced genetic algorithm identification method proposed in this paper for the LuGre model. Section 4 constructs a linear motor platform and outlines its configuration and parameters. It provides a detailed explanation of the parameter identification method for the LuGre friction compensation model and validates the effectiveness of the friction compensation technique as well as the robustness of the designed controller on the physical platform. The performance of the proposed parameter identification algorithm is verified through simulation examples and compared with existing methods. Section 5 concludes the paper and discusses potential future work.

8) Elaborate discussion on friction compensation techniques with reference to literature e.g. 10.1371/journal.pone.0256491 and 10.1371/journal.pone.0258909

Answer:

We thank the reviewer for bringing out these two excellent journal papers, we have elaborated the discussion and it was shown as follows:

Furthermore, Ali K et al. [19] proposes a Fault-Tolerant Control (FTC) strategy for a 5-DoF robotic manipulator, combining an observer-based approach with hardware redundancy to enhance performance under actuator and sensor faults. A dynamic LuGre friction model is derived as the basis for control law design. For FTC, an adaptive back-stepping method is used for fault estimation, while nominal control laws enable controller reconfiguration and observer design. Fault detection compares actual and observed states, followed by fault-tolerant methods using redundant sensors. Results confirm the effectiveness of the proposed FTC strategy with model-based friction compensation, demonstrating improved tracking performance and robustness in the presence

of friction and faults. While Mehmood Y et al. [20] proposes Fractional and Integral Order Fuzzy Sliding Mode Controllers (FSMC, FFSMC) for Skid-Steered Vehicles (SSV) with variable friction coefficients and displaced Center of Gravity (CG). The FFSMC reduces forces from ground-tire interaction during skidding and friction variations. Implemented on a four-wheel SSV during high-speed turns, the controllers were tested in a simulation environment integrating SSV dynamics and a wheel-road model. Tests used a Pioneer-3AT (P-3AT) robot SSV with displaced CG and variable tire friction. Results show the FFSMC achieves reduced state errors and minimal chattering, compensating for wheel response variations due to CG shifts. Additionally, fuzzy tuning minimizes chattering from conventional Sliding Mode Controllers (SMCs).

[19] Ali, K., Mehmood, A., & Iqbal, J. (2021). Fault-tolerant scheme for robotic manipulator — Nonlinear robust back-stepping control with friction compensation. PLoS one, 16(8), e0256491.

[20] Mehmood, Y., Aslam, J., Ullah, N., Alsheikhy, A. A., Din, E. U., & Iqbal, J. (2021). Robust fuzzy sliding mode controller for a skid-steered vehicle subjected to friction variations. PLoS one, 16(11), e0258909.

9) Label Figure 7 so as to convey more useful information.

Answer:

We thank the reviewer for helping improve the quality of the figure, the new figure 7 is labelled and shown as follows:

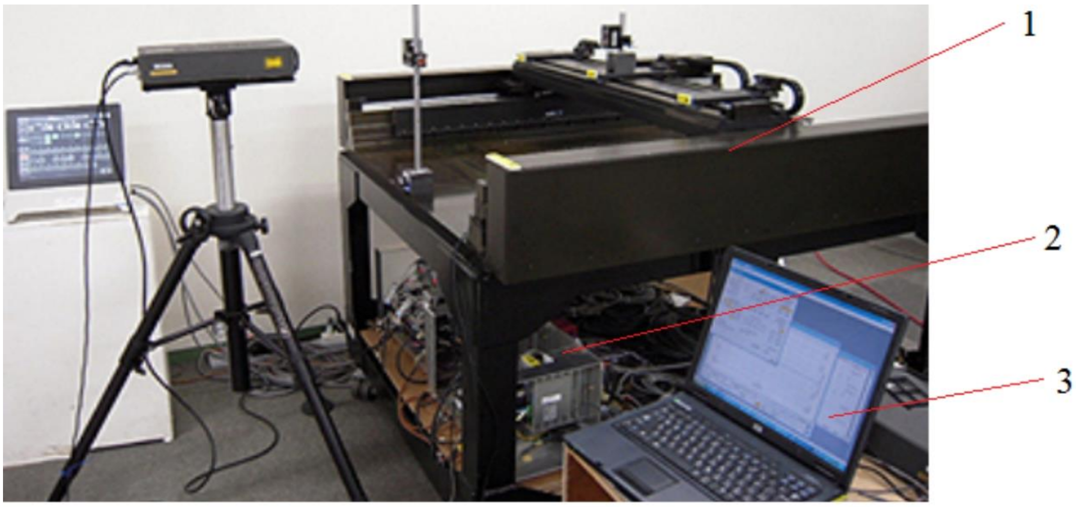

(a) Linear motor precision motion control experimental platform

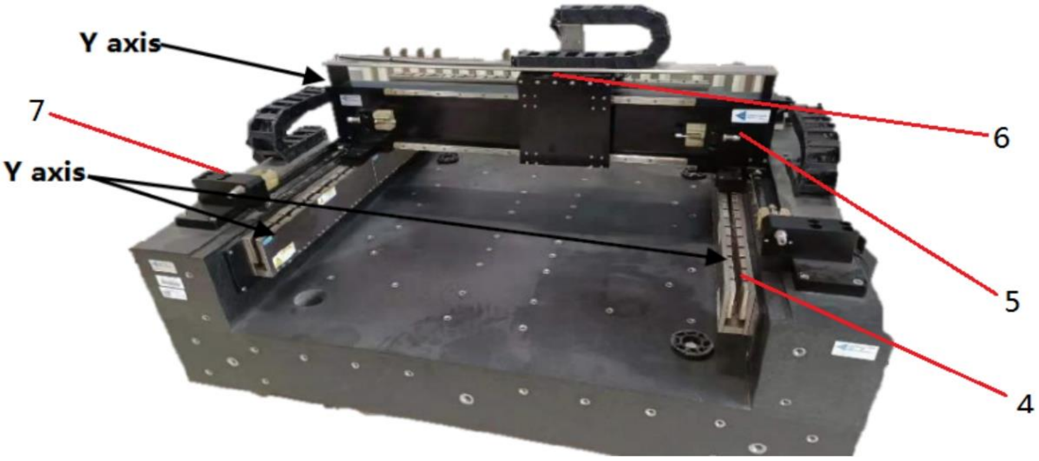

(b) Composition of Longmen linear motor platform

Fig. 7 Physical picture of linear motor platform: 1-Gantry linear motor platform; 2-Linear motor servo drive; 3- industrial control computer.; 4-X-axis grating ruler displacement sensor; 5-X-axis motor; 6- industrial control computer; 7- Y-axis grating ruler displacement sensor.

10) What are the units of coefficients in Table 1?

Answer:

We thank the reviewer for pointing out this, the new Table 1 is shown as follows and all units of coefficients have been added:

Tabel.1 LuGre friction model parameters

Friction force name	Symbol	Unit	Value

Mane stiffness coefficient	σ_0	$(\mathrm{N}\cdot\mathrm{m}/\mathrm{rad})$	242
Mane damping coefficient	σ_1	$(\mathrm{N}\cdot m\cdot s/\mathrm{rad})$	8
Coefficient of viscous friction	σ_2	$(\times 10^{-6} N\cdot\mathrm{m}\cdot\mathrm{s}/\mathrm{rad})$	4.82
Static friction force	F_s	N	1
Stribeck speed	v_s	m/s	0.001
Coulomb friction force	F_c	N	1.5

11) Include results on Step response to precisely characterize the performance of the proposed approach.

Answer:

We thank the reviewer for proposing such a great question, we have worked on this issue and we tried our best to present the new contents:

(2) Control Shear Force and Control Force of Identification Values

For the LuGre model, static parameter identification follows conventional methods, while dynamic parameter identification utilizes sensor outputs (displacement or acceleration) and the control shear force as direct inputs. During identification, the control shear force serves as the target approximation value, simplifying the identification of two dynamic parameters.

For dynamic parameter identification, the servo system's displacement (or acceleration) and control shear force outputs are directly used, with the control shear force acting as the target approximation value. Assuming the dynamic parameter $x_d=\omega_2=[\hat{\sigma}_0 \;\; \hat{\sigma}_1]$ requires identification, the identification error is defined as:

$$e(x_d, t_i)=u(t_i)-u(x_d, t_i) \tag{38}$$

Where, $u(t_i)$ controls the shear force for the servo system, while $u(x_d, t_i)$ model parameter system identifies the output control force.

$$u\left(x_d,t_i\right)=F_f(t)+m\frac{d^2x_i}{dt_i^2} \tag{39}$$

Substituting $z(t)\to z, \dot{z}\to\frac{dz(t)}{d(t)}, \dot{x}\to x$ yields equation:

$$F_f(t)=\hat{\sigma}_0\cdot z+\hat{\sigma}_1\cdot\dot{z}+\hat{\sigma}_2\cdot\dot{x} \tag{40}$$

$$\dot{z}=\dot{x}-\frac{\hat{\sigma}_0}{g(\dot{x})}z \tag{41}$$

The objective function is defined as:

$$J=\frac{1}{2}\sum_{i=1}^{N}e^2\left(y_d,t_i\right) \tag{42}$$

During the identification of power parameters, the servo system outputs $x(t)=0.01\sin(2.2\pi\cdot t)$, and the corresponding identification results for the control force u and friction force are illustrated in Fig. 24. Throughout the identification process, the parameter population search space is defined as $F_c\in[0,100]$, $F_s\in[0,100]$, $v_s\in[0,0.1]$, $\sigma_0\in[0,1000]$, $\sigma_1\in[0,1000]$, $\sigma_2\in[0,50]$.

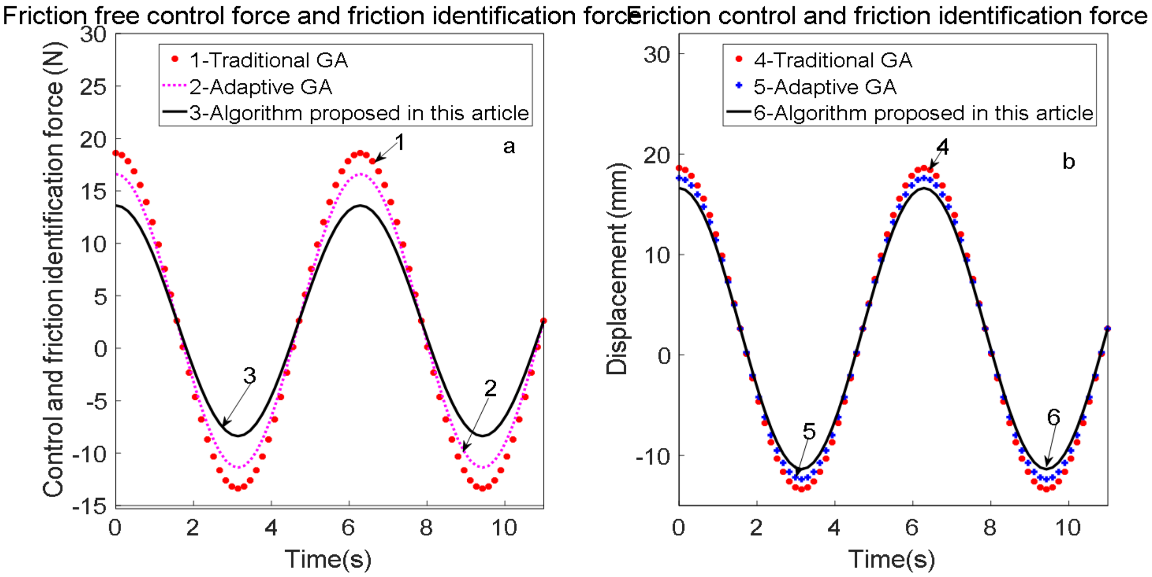

Fig. 24 Control shear force and control force of identification values

It is confirmed that the fuzzy PID control system, integrated with friction feedforward compensation, exhibits both rapid response and steady-state stability. The identification values for friction, control force, and displacement

loop are illustrated in Fig. 25.

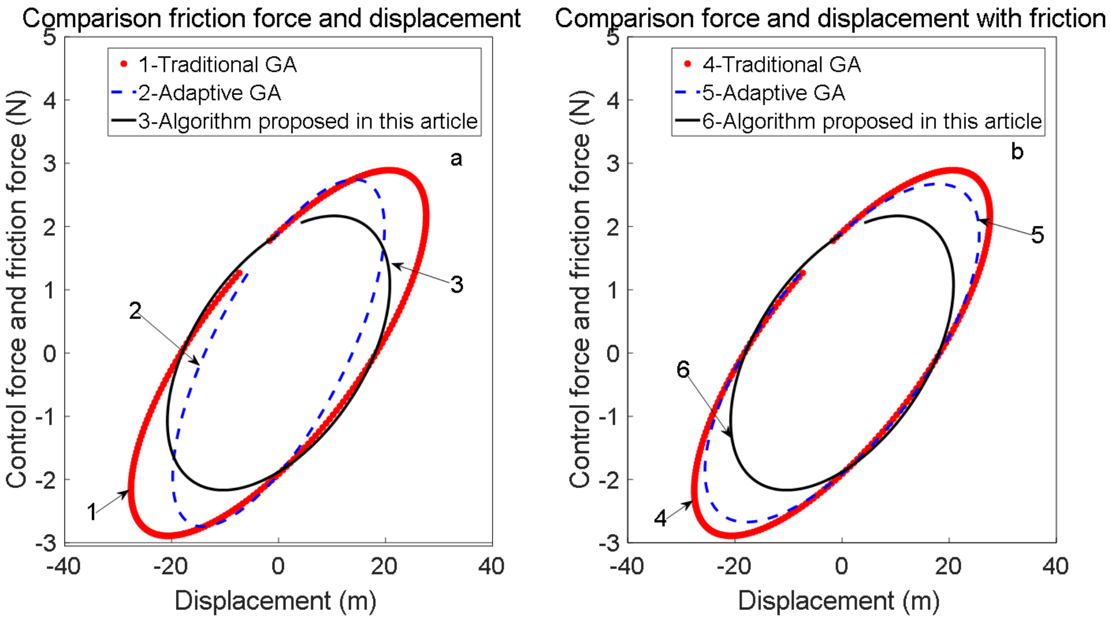

Fig. 25 Friction, control force and displacement loop of identification value

The discrepancy between the friction and control force in the two figures is attributed to inertial force; specifically, the control force is equivalent to the sum of friction and inertial force.

Subsequently, the LuGre friction compensation model was introduced and compared with the simplified model under the same control law, resulting in a significant reduction in tracking error. This fully demonstrates the efficacy of the LuGre friction compensation model.

From the parameter identification results, traditional genetic algorithms (GA), adaptive genetic algorithms, and the proposed algorithm exhibit high accuracy and fast identification speed in determining the static and dynamic nonlinear parameters of the LuGre friction model, establishing them as ideal tools for nonlinear parameter identification.

12) Update the literature review on LuGre model with notable works such as 'Control of an anthropomorphic manipulator using LuGre friction model - Design and experimental validation'

Answer:

We thank the reviewer for pointing out this, we have updated the literature review on LuGre model with the paper mentioned above. The discussion is as follows:

Another work that Ali K et al. [18] proposes is an Integral Sliding Mode Control (ISMC) for an anthropomorphic manipulator using a modified LuGre friction model with friction compensation. His contributions includes deriving a mathematical model for a 5-DOF AUTAREP robotic manipulator, incorporating kinematics, dynamics, and the LuGre friction model, designing a robust controller to compensate for dynamic friction and ensure precise trajectory tracking. The ISMC law, based on the Lyapunov function, addresses system parametric and load torque uncertainties, and implementing the control law on a custom platform with LabVIEW and NI myRIO hardware, including motor drive circuits. However, negative aspect in the proposed technique ISMC with friction compensation is that the temperature effect of joints are not considered as well high control gains and high control efforts related to chattering issues.

13) Section 5.1 presents the simulation results. Please clearly outline the heading for Experimental Results. Are they mentioned in Section 5.2?

Answer:

We deeply appreciate the reviewer for noticing this issue, we have made significant modification to this section and we have outlined the headings for experimental results, the new Section 5.2 are as follows:

5.2 Friction Compensation Experiment

(1) Experimental Study on LuGre Model Feedforward Friction Compensation Controller

To mitigate the adverse effects of nonlinear friction on system performance, a feedforward friction compensation controller based on the LuGre model was designed. The control architecture employs a three-loop strategy, where the current loop is embedded within the motor driver, while the speed and position loops are externally implemented. The controller was developed in Simulink

and deployed via TwinCAT3 software for real-time execution.

The compensation scheme utilizes feedback speed as the input to the LuGre friction model. To address sampling-period delays inherent in feedback-based compensation, a feedforward friction compensation structure (Fig. 21) was adopted. This structure directly injects the estimated friction force into the control input, bypassing latency issues and enhancing system responsiveness.

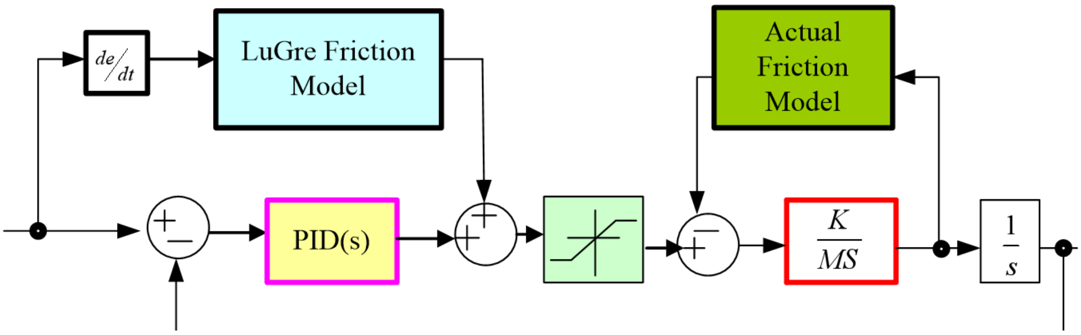

Fig. 21 Feedforward friction compensation model

By introducing a sinusoidal signal $x(t)=15[\cos(\pi t)-\pi/6]$ into both control systems, the signal tracking performance is analyzed. The experiments are divided into two groups based on the presence or absence of feedforward compensation for comparative analysis. The speed tracking curves before and after friction compensation are illustrated in Fig. 22.

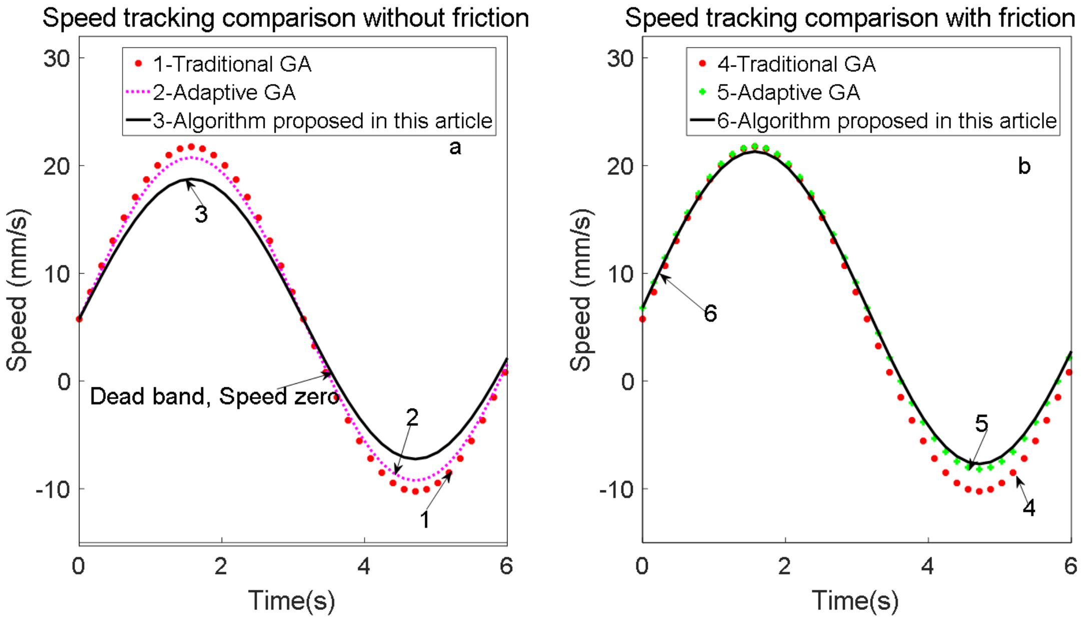

Fig. 22 Speed tracking curve before and after friction compensation

As illustrated in the speed tracking results, a 'dead zone' is observed during zero-speed transitions. The implementation of friction feedforward compensation significantly mitigates this issue, achieving minimal position tracking error and smooth velocity transitions at the zero-crossing point.

The position tracking curves before and after the application of friction compensation are illustrated in Fig. 23.

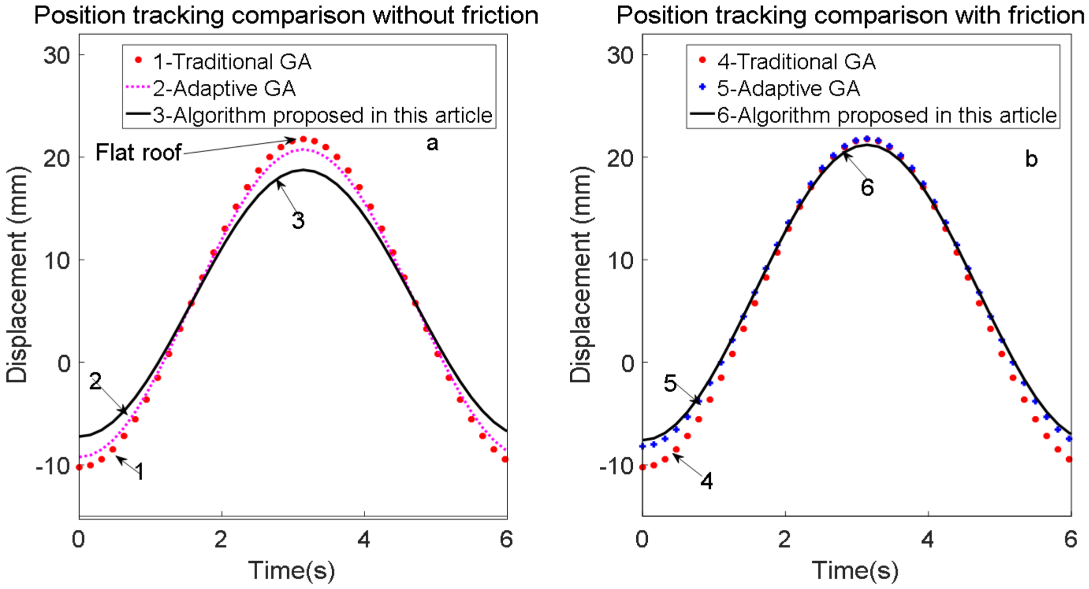

Fig. 23 Position tracking curve before and after friction compensation

The experimental results demonstrate that feedforward friction compensation control based on the LuGre model significantly outperforms traditional PID control in addressing speed tracking imbalances caused by nonlinear friction. This improvement enhances both speed and position tracking accuracy, showcasing greater practical applicability in engineering contexts.

(2) Control Shear Force and Control Force of Identification Values

For the LuGre model, static parameter identification follows conventional methods, while dynamic parameter identification utilizes sensor outputs (displacement or acceleration) and the control shear force as direct inputs. During identification, the control shear force serves as the target approximation value, simplifying the identification of two dynamic parameters.

For dynamic parameter identification, the servo system's displacement (or acceleration) and control shear force outputs are directly used, with the control

shear force acting as the target approximation value. Assuming the dynamic parameter $x_d = \omega_2 = [\hat{\sigma}_0 \quad \hat{\sigma}_1]$ requires identification, the identification error is defined as:

$$e(x_d, t_i) = u(t_i) - u(x_d, t_i) \tag{38}$$

Where, $u(t_i)$ controls the shear force for the servo system, while $u(x_d, t_i)$ model parameter system identifies the output control force.

$$u(x_d, t_i) = F_f(t) + m\frac{d^2 x_i}{dt_i^2} \tag{39}$$

Substituting $z(t) \to z, \dot{z} \to \frac{dz(t)}{d(t)}, \dot{x} \to x$ yields equation:

$$F_f(t) = \hat{\sigma}_0 \cdot z + \hat{\sigma}_1 \cdot \dot{z} + \hat{\sigma}_2 \cdot \dot{x} \tag{40}$$

$$\dot{z} = \dot{x} - \frac{\hat{\sigma}_0}{g(\dot{x})} z \tag{41}$$

The objective function is defined as:

$$J = \frac{1}{2}\sum_{i=1}^{N} e^2(y_d, t_i) \tag{42}$$

During the identification of power parameters, the servo system outputs $x(t) = 0.01\sin(2.2\pi \cdot t)$, and the corresponding identification results for the control force u and friction force are illustrated in Fig. 24. Throughout the identification process, the parameter population search space is defined as $F_c \in [0,100]$, $F_s \in [0,100]$, $v_s \in [0,0.1]$, $\sigma_0 \in [0,1000]$, $\sigma_1 \in [0,1000]$, $\sigma_2 \in [0,50]$.

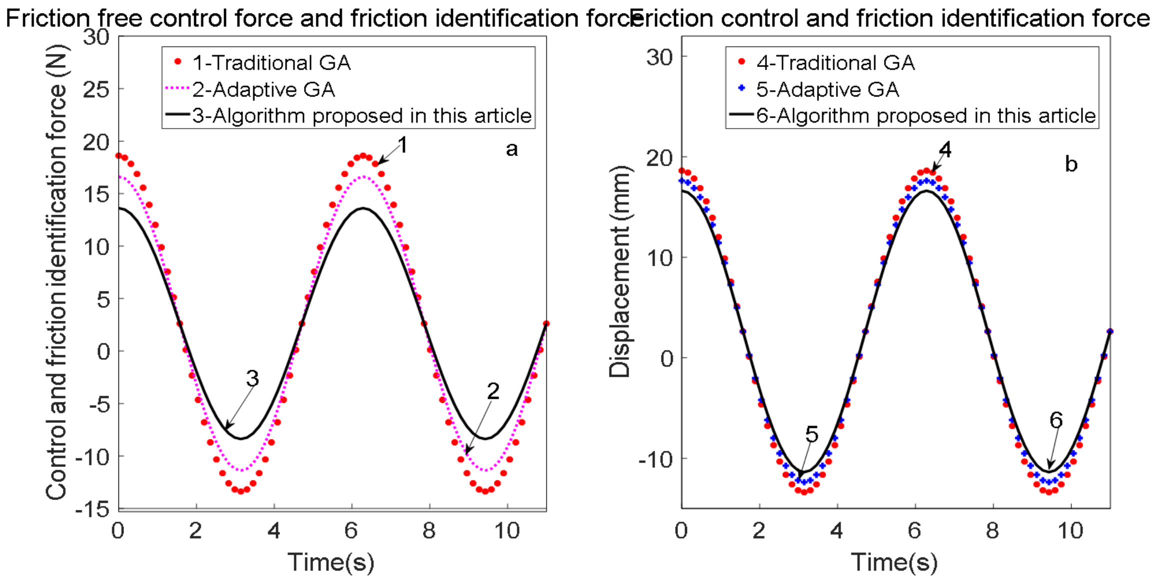

Fig. 24 Control shear force and control force of identification values

It is confirmed that the fuzzy PID control system, integrated with friction feedforward compensation, exhibits both rapid response and steady-state stability. The identification values for friction, control force, and displacement loop are illustrated in Fig. 25.

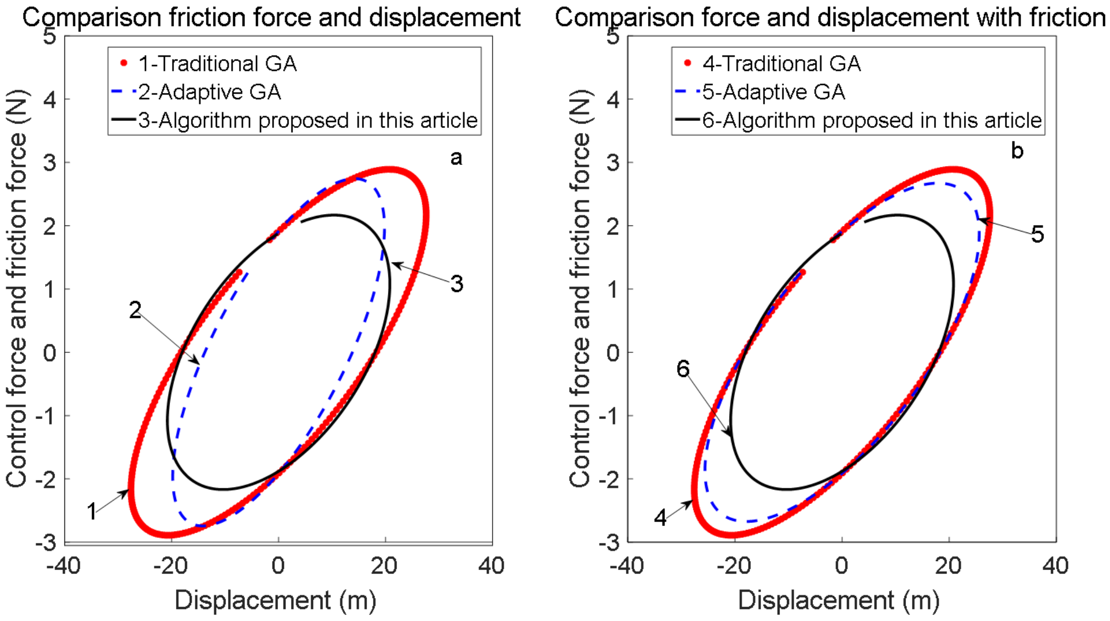

Fig. 25 Friction, control force and displacement loop of identification value

The discrepancy between the friction and control force in the two figures is attributed to inertial force; specifically, the control force is equivalent to the sum of friction and inertial force.

Subsequently, the LuGre friction compensation model was introduced and compared with the simplified model under the same control law, resulting in a significant reduction in tracking error. This fully demonstrates the efficacy of the LuGre friction compensation model.

From the parameter identification results, traditional genetic algorithms (GA), adaptive genetic algorithms, and the proposed algorithm exhibit high accuracy and fast identification speed in determining the static and dynamic nonlinear parameters of the LuGre friction model, establishing them as ideal tools for nonlinear parameter identification.

14) Also, the results need more critical and conclusive discussion. Moreover, please include limitations of the presented study.

Answer:

We thank the reviewer for improving the quality of the manuscript, we have added critical and conclusive discussion as well as limitations:

The study employed the parameter identification method for the LuGre friction compensation model, and the efficacy of the friction compensation technique along with the robustness of the designed controller were substantiated on a physical testbed.

Building upon the LuGre friction model and an enhanced adaptive genetic identification algorithm, the findings of this research are anticipated to mitigate the adverse effects of nonlinear friction on high-precision and ultra-low-speed servo systems. Nonetheless, constrained by various factors, the proposed method's performance has been initially corroborated only within a simulation framework. Prospective research endeavors will concentrate on the following domains:

We aim to implement the algorithm delineated in this study within actual high-precision and ultra-low-speed servo systems to ascertain its performance in real-world engineering scenarios, and to further refine the algorithm adaptively to tackle emergent challenges.

Although the convergence velocity of the algorithm presented herein surpasses that of the adaptive genetic algorithm, it still lags behind the conventional genetic algorithm. Enhancing the proposed algorithm to accelerate convergence and enhance its suitability for engineering applications remains a pivotal objective for future investigations.

15) Please thoroughly proofread the paper for typos and linguistic improvements.

Answer:

We thank the reviewer for proposing constructive comments improving the quality of the manuscript, we have sought help from professionals and thoroughly proofread the paper for typos and made linguistic improvements.

Reviewer 3

Comment 1: In abstract and also in the introduction there were some phrases that was repeated similarly, it's better to have a different type of explanation and more summarized in abstract.

Answer:

We thank the reviewer for pointing out our issue, we have rewritten abstract as well as introduction to avoid some phrases repeated similarly, also, the abstract is summarized shown as follows:

Nonlinear friction in high-precision, ultra-low-speed servo systems severely degrades performance, causing low-speed crawling, static errors, and limit-cycle oscillations. This study introduces the LuGre friction model to describe these phenomena mathematically and proposes an improved genetic algorithm (GA) for precise parameter identification. Simulations demonstrate that LuGre-based feedforward compensation outperforms conventional proportional-integral-derivative (PID) control, effectively mitigating speed tracking errors and enhancing both speed and position accuracy. Experimental validation on a linear motor platform confirms the method's efficacy, achieving a 25.1% improvement in tracking accuracy. The results highlight the practical relevance of this approach for precision servo systems.This work has achieved a practical identification framework for LuGre parameters, combining GA optimization with transient/steady-state data, feedforward compensation that directly injects estimated friction forces, bypassing feedback delays and Experimental verification of the method's industrial applicability, with code and datasets openly shared for reproducibility.

Comment 2: It is better to also have an explanation on how to calculate the target friction force based on motor dynamic equations.

Answer:

We thank the reviewer for improving the quality of the manuscript. We have

added a new section discussing the calculation of friction force based on motor dynamic equations.

4.1 Calculation of Target Frictional Force via Motor Dynamics

The general mechanical dynamics model for the X-axis of a linear synchronous motor (LSM) servo system is expressed as:

$$M_x \cdot \ddot{y} + C_x \cdot \dot{y} + K_x \cdot y = -F_{rx} + F_x + F_{dis} \tag{23}$$

Where y, $\dot{y}$ and $\ddot{y}$ denote the displacement, velocity, and acceleration of the LSM, respectively. M_x, C_x and K_x represent the moving mass, equivalent viscous damping coefficient, and stiffness constant of the X-axis. $F_x = K_F \cdot K_u \cdot u_x$ is the electromagnetic driving force (K_F : force constant; K_u : amplifier gain; u_x : control input), F_{rx} is the friction force, and F_{dis} encompasses unmodeled disturbances and external interference.

By neglecting additional external characteristics of the linear motor, the simplified nonlinear dynamics model of the X-axis reduces to:

$$M_x \cdot \ddot{y} = -F_{rx} + F_x + F_{dis} \tag{24}$$

Here M is the load mass; $F = K \cdot u$ is the electromagnetic driving force($K = K_F \cdot K_u$: lumped gain), F_r is the frictional force. Equation (24) describes the rigid-body dynamics of the motor as a second-order system with control voltage u as the input and displacement y as the output:

$$M \cdot \ddot{y} = -F_r + K \cdot u + F_{dis} \tag{25}$$

The LuGre friction model characterizes F_r using stiffness coefficient σ_0, damping coefficient σ_1, and average bristle deformation z :

$$F_r = \sigma_0 \cdot z + \sigma_1 \cdot \dot{z} + \sigma_2 \cdot \dot{x}(t) \tag{26}$$

Under pre-sliding conditions (i.e., no macroscopic motion, $z \approx x$ and $\dot{z} \approx \dot{x}(t)$), the friction force simplifies to:

$$F_r \approx \sigma_0 \cdot x + \sigma_1 \cdot \dot{x} + \sigma_2 \cdot \dot{x} \tag{27}$$

That yields:

$$M \cdot \ddot{x} = -\left(\sigma_0 \cdot x + \sigma_1 \cdot \dot{x} + \sigma_2 \cdot \dot{x}\right) + K \cdot u + F_{dis} \tag{28}$$

Ignoring $F_{dis} = 0$, the simplified equation is described as:

$$M \cdot \ddot{x} = -\left(\sigma_0 \cdot x + \sigma_1 \cdot \dot{x} + \sigma_2 \cdot \dot{x}\right) + K \cdot u \tag{29}$$

Simplify the equation into an expression:

$$M \cdot \ddot{x} + \sigma_0 \cdot x + \sigma_1 \cdot \dot{x} + \sigma_2 \cdot \dot{x} = K \cdot u \tag{30}$$

The input is defined as the control voltage u, and the output is the motor displacement x. Applying the Laplace transform yields the transfer function:

$$\frac{X(s)}{U(s)} = \frac{K}{Ms^2 + \left(\sigma_1 + \sigma_2\right)s + \sigma_0} \tag{31}$$

It is evident that when the system is actuated by electromagnetic driving forces and remains in the pre-sliding regime (i.e., prior to macroscopic motion), its dynamics can be approximated as a linear second-order damped system. Let the natural frequency be denoted as ω_n and the damping ratio as ξ. The governing equation can then be expressed in the canonical form of a second-order system:

$$\frac{X(s)}{U(s)} = \frac{K}{\sigma_0} \cdot \frac{\omega_n^2}{s^2 + 2\xi \cdot \omega_n \cdot s + \omega_n^2} \tag{32}$$

Where, the expressions for the natural frequency ω_n and damping coefficient ξ are described as:

$$\omega_n = \sqrt{\sigma_0 / M} \tag{33}$$

$$\xi = \frac{\sigma_1 + \sigma_2}{2\sqrt{\sigma_0 / M}} \tag{34}$$

From the knowledge of control theory, it can be concluded that:

$$x_{ss} = \frac{K}{\sigma_0} I \tag{35}$$

$$M_p = e^{-\frac{\xi\pi}{\sqrt{1-\xi^2}}} \times 100\% \tag{36}$$

Where, x_{ss} is the steady-state value of step response; M_p is the overshoot of the response.

Consequently, under steady-state conditions, a step control voltage input may be introduced to the linear motor system to record its step response. The dynamic parameters of the system can then be systematically identified through analysis of the steady-state displacement and percentage overshoot derived from the transient response profile.

As illustrated in Figure 12, the step response of the stationary motor under a control voltage excitation of $u = 0.1\text{V}$ exhibits a steady-state displacement of $x_{ss} = 2.737 \times 10^{-4}$ and a percentage overshoot of $M_p = 43.16\%$. The dynamic parameters of the LuGre friction compensation model are computed as $\sigma_0 = 2421.525\text{N} \cdot \text{m/rad}$ and $\sigma_1 = 181.525\text{N} \cdot m \cdot s/\text{rad}$. These values quantitatively characterize the pre-sliding stiffness and viscous damping properties of the frictional interface.

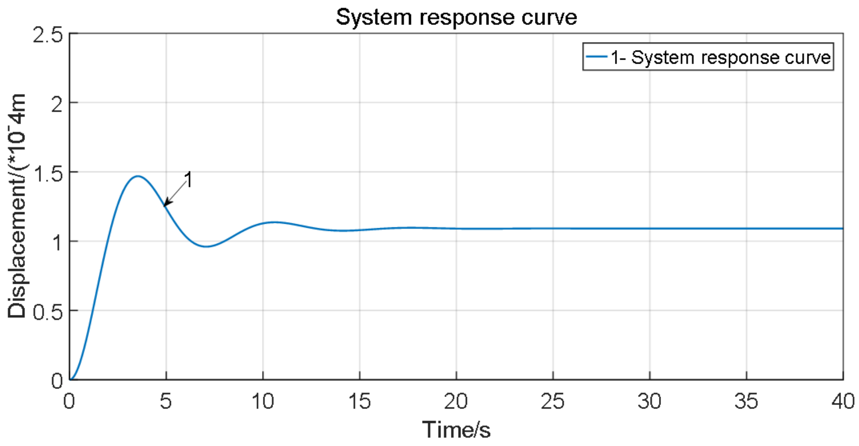

Fig. 12 System response curve

Comment 3: The arrows in Fig.2 could have been shown more understandable.

Answer:

We thank the reviewer for pointing out this issue, we have changed Figure 2 and fully described using variables given in the article:

In Equation (7) and (8), $u(k)$ is the input of the system; $F_{fr}(k)$ is the actual observed value of the system output, and $F_{fr}(k)$ represents the LuGre target

friction force; $\hat{F}_{fl}(\hat{\vartheta}, \dot{x}(k))$ denotes the estimated model calculation value, which is the LuGre friction force calculated from the identified parameters during the iterative process; $e(k)$ is the estimation error, $e(k) = F_{fr}(k) - \hat{F}_{fl}(\hat{\vartheta}, \dot{x}(k))$. The fundamental principles of system identification are illustrated in Figure 2.

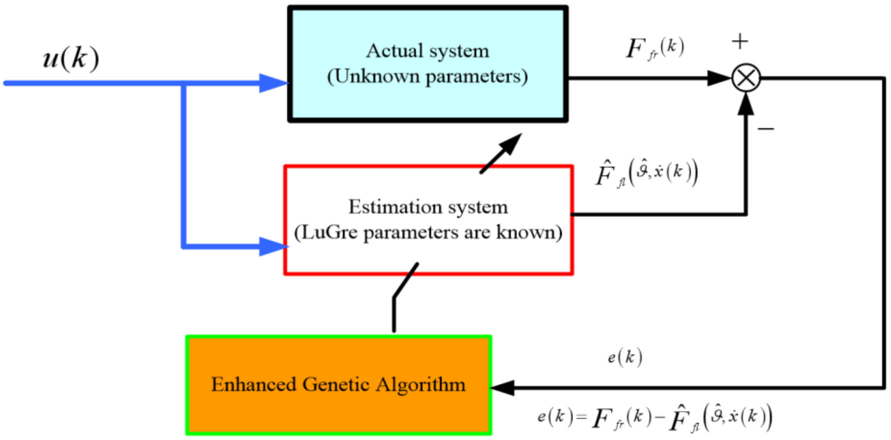

Fig. 2 Basic principles of system identification

Comment 4: Having a comparation between suggested controller and another type to display the improvement and efficiency.

Answer:

We thank the reviewer for pointing out this issue, we have added the following contents in the manuscript to reflect the effectiveness of the improved genetic algorithm compared with the traditional algorithm:

The convergence process of the Genetic Algorithm for LuGre parameter identification is shown in Figure 5.

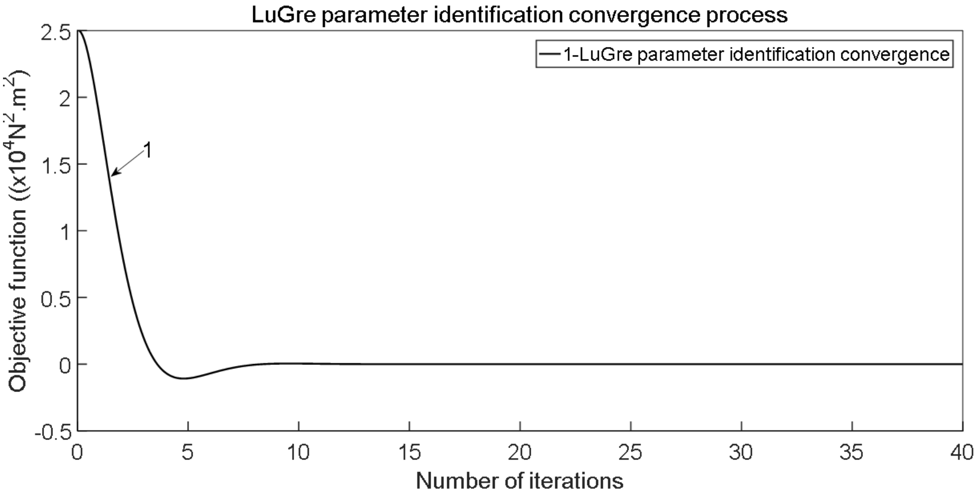

Fig. 5 LuGre parameter identification convergence process

From Figure 5, it can be clearly observed that in the initial stages of the genetic process, the convergence speed is relatively fast. As the number of generations increases, the convergence tends to get trapped in a local optimum, slowing down the speed. When the number of generations reaches a certain level, the Genetic Algorithm escapes the local optimum and gradually converges to the global optimum. Comparison curve of objective function (J_k) is shown in Fig.6.

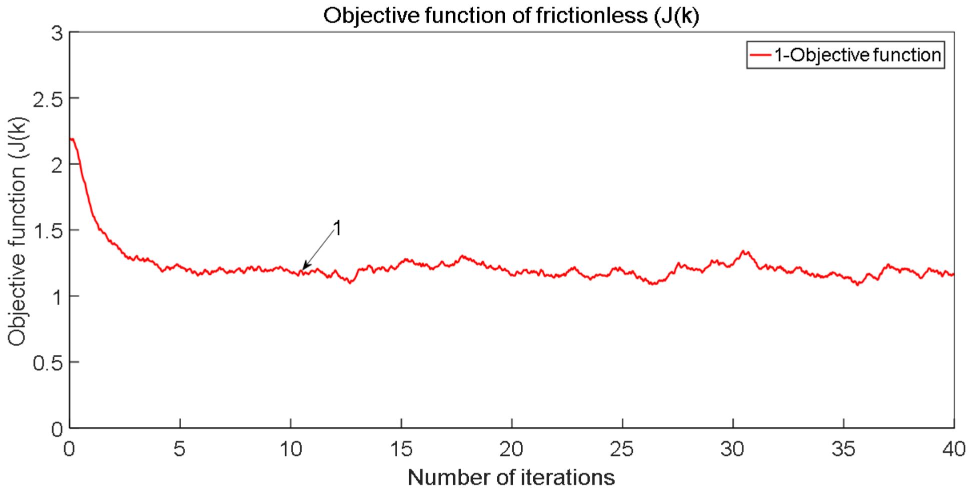

Fig. 6 Comparison curve of objective function (J_k)

The comparative results among the traditional genetic identification algorithm, the adaptive genetic identification algorithm, and the algorithm proposed in this study, along with the corresponding objective function value curves, are illustrated in Figure 7.

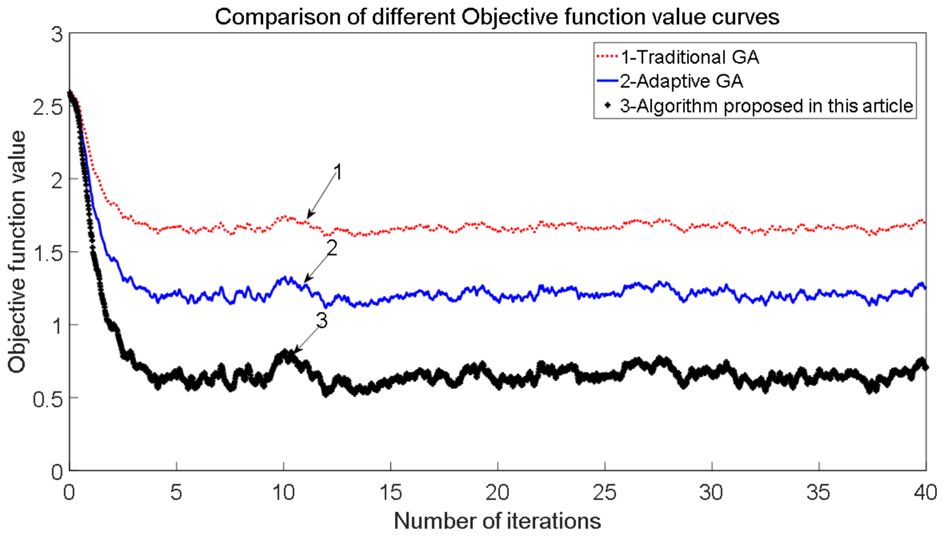

Fig. 7 Comparison of different Objective function value curves

Figures 6 and 7 present a comparison of the traditional genetic identification algorithm, the adaptive genetic identification algorithm, and the algorithm proposed in this paper, along with the corresponding objective function value curves. The results demonstrate that the identified friction force closely aligns with the model, exhibiting minimal error. However, in cases where the identified maximum static friction force significantly exceeds the model's value, this discrepancy may be attributed to testing errors in the provided data. Although some data points fall outside the expected range, they have not been excluded from the analysis. In comparison to the other algorithms, the proposed algorithm in this study achieves faster and more accurate identification of the friction model parameters.

Comment 5: Mentioning the strength and week points of each method used, in introduction.

Answer:

We have completely rewritten the induction to mention the strength and weak point of each method used, the introduction is as follows:

“Ali K et al. [17] proposed an advanced control algorithm that combines fast integral terminal sliding mode control, robust exact differentiator, and feedforward neural network-based estimator. Firstly, considering the dynamic

LuGre friction compensation model, a mechanical manual model of an n-degree-of-freedom system was established. Then, a friction compensation based FIT-SMC nonlinear control method was proposed, which showed better performance than traditional compensators. **Also, it is pointed out** by author that it is a computationally complicated function that takes a long time to execute."

"Another work that Ali K et al. [18] proposes is an Integral Sliding Mode Control (ISMC) for an anthropomorphic manipulator using a modified LuGre friction model with friction compensation. His contributions includes deriving a mathematical model for a 5-DOF AUTAREP robotic manipulator, incorporating kinematics, dynamics, and the LuGre friction model, designing a robust controller to compensate for dynamic friction and ensure precise trajectory tracking. The ISMC law, based on the Lyapunov function, addresses system parametric and load torque uncertainties, and implementing the control law on a custom platform with LabVIEW and NI myRIO hardware, including motor drive circuits. **However,** negative aspect in the proposed technique ISMC with friction compensation is that the temperature effect of joints are not considered as well high control gains and high control efforts related to chattering issues."

"Aguilar Avelar et al. [26] first discussed the modeling, identification, and compensation of the influence of nonlinear friction on flywheel control. A new algorithm for stabilizing the flywheel in an unstable position and a friction compensation controller based on feedback linearization have been proposed. The experimental results show that the established asymmetric Coulomb friction element dynamic model can better characterize the real experimental platform of the system, with good stability and low power consumption control performance. **However**, the velocity of the pendulum s slightly greater for the proposed algorithm."

"Li et al. [27] proposed an improved Stribeck friction model (SFM) and an optimization algorithm consistent with the positioning platform, and verified a compensator based on the friction model and disturbance observer through

simulation. The simulation results show that the compensator based on friction model with added disturbance has better performance than traditional compensators. In addition, compensation comparisons were made between Coulomb friction, traditional SFM, and improved SFM. The experimental results showed that compared with Coulomb friction compensation, the improved SFM compensation reduced the following errors by 67.67% and 51.63% at speeds of 0.005m/s and 0.05m/s, respectively. **However**, the proposed modified SFM has a limitation in terms of velocity reversals."

"Han et al. [28] established a robust positioning control scheme using a friction parameter observer and a recursive fuzzy neural network based on sliding mode control. The friction state observer fully captures nonlinear dynamic friction, and uses recursive fuzzy neural network technology to establish an approximation method for system uncertainty, improving positioning accuracy. The performance of the control scheme is verified through experiments. **However**, for the purpose of more enhancement of precision positioning, the authors consider the robust control using the RFNN estimator and adaptive approximation error estimator."

"Meng et al. [29] constructed an adaptive robust controller for dynamic friction compensation based on the LuGre friction compensation model. Adopting online recursive least squares estimation for model parameter identification, achieving real-time parameter updates, and designing a sliding mode controller to alleviate the degradation of control performance caused by parameter estimation errors and unmodeled dynamics. **It is noted that** since the trajectory is not always persistently exciting, the parameter estimates exhibit slow convergence."

"In Jin et al.'s work [30], a complementary sliding mode control method based on approximation angle saturation function is proposed, which can reduce the boundary layer as the state trajectory changes until it converges to the sliding surface. This method effectively eliminates system chattering and improves position tracking accuracy and robustness. The designed control method was

compared with the controller using traditional saturation function, and the results showed that the control method can achieve lower tracking error and stronger robustness. **However**, it can only be applied to the case where the trajectory of the state moves towards the switching surface, also the friction force is considered as a part of the lumped uncertainty, and the influence of friction force on the system is not properly analyzed."

"Chen et al. [32] proposed a discrete adaptive sliding mode controller for high-precision motion control of linear permanent magnet synchronous motors, which achieves real-time gain tuning capability and robustness, ensuring the performance of tracking tasks. Experimental results show that the designed controller has fast response and strong robustness to uncertainty and noise. **However**, maximum static friction force may arouse a higher tracking error at the beginning of the move."

"Zhao et al. [33] proposed an adaptive neural network nonsingular fast terminal sliding mode control for high-precision position tracking of permanent magnet synchronous motors in the presence of uncertainty. Nonsingular fast terminal sliding mode control was used to achieve rapid convergence of the system at the equilibrium point. **However**, the approach often inadequately address parameter uncertainty, compromising the quality of identification outcomes"

Comment 6: Good breakdown of parameters to identify.

Answer:

We thank the reviewer for proposing such a great suggestion, We have added a new section in Section 1 in order to give a good breakdown of parameter to identify:

1.3 Static and Dynamic Friction to be Identified

(a) Identification of static friction parameters

When the system is running at a constant and uniform speed, the acceleration of the system is 0, and $\frac{dz}{dt}=0$ can be obtained, then the frictional force can be

obtained at this time.

The expression for the friction force F_r during steady-state motion varies with the speed change, and is as follows:

$$\begin{aligned} F_r &= \sigma_0 \cdot z_s + \sigma_2 \cdot \dot{x}(t) = g(\dot{x}(t)) \cdot z_s + \sigma_2 \cdot \dot{x}(t) \\ &= \left[F_c + (F_s - F_c) \cdot e^{-(\dot{x}(t)/v_s)^2} \right] \cdot \operatorname{sgn}(\dot{x}(t)) + \sigma_2 \cdot \dot{x}(t) \end{aligned} \quad (5)$$

According to the formula above, the parameter vector to be identified is set as follows:

$$\omega_1 = \left[\hat{F}_c \quad \hat{F}_s \quad \hat{v}_s \quad \hat{\sigma}_2 \right] \quad (6)$$

In Equation (6), ω_1 is static friction parameter vector to be identified.

From the above formula (6), it can be seen that the LuGre friction model is clear and concise. Compared with other models, this model can smoothly transition from the current friction state to another friction state and has good continuity to a certain extent. In addition, this model can accurately express complex nonlinear friction characteristics such as Coulomb friction, viscous friction, friction hysteresis, Stribeck effect, and friction memory. And the unknown parameters can be obtained through parameter identification methods, providing possibilities for the design and application of various friction compensation algorithms. The relevant parameters that need to be set in the genetic algorithm will be provided in the simulation section later. Combining the above derived statements, the static friction parameters of the LuGre friction model can be obtained.

(b) Identification of dynamic friction parameters

The optimal result obtained by identifying the static friction parameters will serve as the basis for identifying the dynamic friction parameters. Dynamic friction parameter identification can be carried out using the acceleration and control force output by the system. The parameter vector to be identified is set as follows:

$$\omega_2 = \left[\hat{\sigma}_0 \quad \hat{\sigma}_1 \right] \quad (8)$$

In Equation (8), ω_2 is Dynamic friction parameter vector to be identified. Combining the LuGre friction model formula, there are:

$$z = \dot{x}(t) - \sigma_0 \cdot \frac{\dot{x}(t)}{g(\dot{x}(t))} z \tag{9}$$

$$F_r = \hat{\sigma}_0 \cdot z + \hat{\sigma}_1 \cdot \dot{z} + \sigma_2 \cdot \dot{x}(t) \tag{10}$$

The relevant parameters that need to be set in the genetic algorithm will be provided in the simulation section later. By combining the above formula, the dynamic friction parameters of the LuGre friction model can be obtained.

Comment 7: Defining the LuGre equations in full term and not simplifying it.

Answer:

We thank the reviewer for proposing such a good suggestion, the LuGre friction model is a comprehensive and widely used dynamic friction model that captures the complex behavior of friction in mechanical systems, it is a dynamic friction model that integrates the Dahl model with steady-state friction characteristics to comprehensively describe friction phenomena such as the Stribeck effect, presliding displacement, hysteresis, and frictional lag. By incorporating the Stribeck effect, this model captures the variable friction behavior that occurs at extremely low velocities, enhancing its ability to simulate real-world friction dynamics.

The model defines friction force $F_f(t)$ as a combination of bristle deflection dynamics and velocity-dependent terms.

1. Friction force:

$$F_f(t) = \sigma_0 \cdot z(t) + \sigma_1 \cdot \frac{dz(t)}{d(t)} + \sigma_2 \cdot \dot{x}(t) \quad (2)$$

- σ_0: Bristle stiffness coefficient (N/m).
- σ_1: Bristle damping coefficient (N·s/m).

- σ_2: Viscous friction coefficient (N·s/m).
- $z(t)$: Average bristle deflection (m).
- $\dot{x}(t)$: Relative velocity (m/s).

2. Bristle Deflection Dynamics:

$$\frac{dz(t)}{d(t)} = \dot{x}(t) - \frac{\sigma_0}{g(\dot{x}(t))} z(t) \cdot |\dot{x}(t)| \quad (3)$$

The function $g(\dot{x}(t))$ models the Stribeck effect:

$$g(\dot{x}(t)) = F_c + (F_s - F_c) \cdot e^{-(\dot{x}(t)/v_s)^2} \quad (4)$$

- F_c: Coulomb friction (N).
- F_s: Static friction (N).
- v_s: Stribeck velocity (m/s).

Comment 8: In introduction it is better to focus on evaluated of LuGre model instead of mentioning the history of this method.

Answer:

We have rewritten the introduction, it now focuses on evaluation of LuGre model rather than mentioning the history, it can be seen as follows:

Friction is a critical factor influencing the performance of electromechanical servo systems. To enhance system performance, appropriate friction compensation methods must be employed to mitigate or eliminate the adverse effects of friction [1-3]. It is essential to address nonlinear frictional forces for optimal servo system functionality [4].

Among the various friction compensation methodologies, those based on friction models are particularly effective as they can deliver swift and accurate compensation, making them a widely adopted approach. In terms of reducing the impact of friction, compensating for nonlinear friction is more practical and cheaper than pure mechanical methods, such as improving contact surface

materials and lubrication conditions [5-7].

In order to compensate for nonlinear friction, the key step is to establish an accurate friction model and accurately determine the parameters of the model, which is helpful for conducting friction compensation research in precision mechanical system engineering applications, such as trajectory tracking and dynamic prediction. So far, many effective mathematical models have been proposed to describe friction characteristics, such as Coulomb model, Stribeck model, Dahl model, Leuven model, LuGre model, etc [7]. Among them, the LuGre friction model is a relatively complete friction model that can accurately describe the characteristics of viscous sliding motion, friction hysteresis, pre sliding displacement, and variable maximum static friction force during the friction process [8]. During low-speed operation of the motor, negative phenomena such as low-speed crawling, unstable operation, and limit cycles are particularly prone to occur, all of which are caused by nonlinear friction [9-10].

There are two main types of friction compensation methods [11-15]. The first type is compensation that is not based on friction models, treating friction as a disturbance and suppressing its effects by providing the system with anti-interference capabilities.

Zhao et al. [16] studied a novel sliding mode control algorithm with adaptive gain, introduced an integral term to design the sliding surface, and selected a torsion control algorithm based on high-order SMC idea as the switching control law. Ali K et al. [17] proposed an advanced control algorithm that combines fast integral terminal sliding mode control, robust exact differentiator, and feedforward neural network-based estimator. Firstly, considering the dynamic LuGre friction compensation model, a mechanical manual model of an n-degree-of-freedom system was established. Then, a friction compensation based FIT-SMC nonlinear control method was proposed, which showed better performance than traditional compensators. Also, it is pointed by author that it is a computationally complicated function that takes a long time to execute.

Another work that Ali K et al. [18] proposes is an Integral Sliding Mode Control (ISMC) for an anthropomorphic manipulator using a modified LuGre friction model with friction compensation. His contributions includes deriving a mathematical model for a 5-DOF AUTAREP robotic manipulator, incorporating kinematics, dynamics, and the LuGre friction model, designing a robust controller to compensate for dynamic friction and ensure precise trajectory tracking. The ISMC law, based on the Lyapunov function, addresses system parametric and load torque uncertainties, and implementing the control law on a custom platform with LabVIEW and NI myRIO hardware, including motor drive circuits. However, negative aspect in the proposed technique ISMC with friction compensation is that the temperature effect of joints are not considered as well high control gains and high control efforts related to chattering issues.

Furthermore, Ali K et al. [19] proposes a Fault-Tolerant Control (FTC) strategy for a 5-DoF robotic manipulator, combining an observer-based approach with hardware redundancy to enhance performance under actuator and sensor faults. A dynamic LuGre friction model is derived as the basis for control law design. For FTC, an adaptive back-stepping method is used for fault estimation, while nominal control laws enable controller reconfiguration and observer design. Fault detection compares actual and observed states, followed by fault-tolerant methods using redundant sensors. Results confirm the effectiveness of the proposed FTC strategy with model-based friction compensation, demonstrating improved tracking performance and robustness in the presence of friction and faults. While Mehmood Y et al. [20] proposes Fractional and Integral Order Fuzzy Sliding Mode Controllers (FSMC, FFSMC) for Skid-Steered Vehicles (SSV) with variable friction coefficients and displaced Center of Gravity (CG). The FFSMC reduces forces from ground-tire interaction during skidding and friction variations. Implemented on a four-wheel SSV during high-speed turns, the controllers were tested in a simulation environment integrating SSV dynamics and a wheel-road model. Tests used a Pioneer-3AT (P-3AT) robot SSV with displaced CG and variable tire friction. Results show the FFSMC

achieves reduced state errors and minimal chattering, compensating for wheel response variations due to CG shifts. Additionally, fuzzy tuning minimizes chattering from conventional Sliding Mode Controllers (SMCs). P. Moradi et al. as well as M. H. Korayem et al. has also performed similar work and achieved significant findings [21-24].

Cong [25] designed an improved Stribeck friction model. Based on the improved model, an inertia stabilization platform and speed stabilization loop feedforward control were designed in the closed-loop control system to avoid vibration and limit cycle problems caused by changes in motion direction and excessive friction compensation. The experimental results show that, with the use of nonlinear friction model compensation, the isolation performance of both the tracking system and the isolation system is superior to the corresponding control system.

Aguilar Avelar et al. [26] first discussed the modeling, identification, and compensation of the influence of nonlinear friction on flywheel control. A new algorithm for stabilizing the flywheel in an unstable position and a friction compensation controller based on feedback linearization have been proposed. The experimental results show that the established asymmetric Coulomb friction element dynamic model can better characterize the real experimental platform of the system, with good stability and low power consumption control performance. However, the velocity of the pendulum s slightly greater for the proposed algorithm.

Li et al. [27] proposed an improved Stribeck friction model (SFM) and an optimization algorithm consistent with the positioning platform, and verified a compensator based on the friction model and disturbance observer through simulation. The simulation results show that the compensator based on friction model with added disturbance has better performance than traditional compensators. In addition, compensation comparisons were made between Coulomb friction, traditional SFM, and improved SFM. The experimental results showed that compared with Coulomb friction compensation, the improved SFM

compensation reduced the following errors by 67.67% and 51.63% at speeds of 0.005m/s and 0.05m/s, respectively. However, the proposed modified SFM has a limitation in terms of velocity reversals. Fixed model friction compensation includes a fixed friction model compensation term, which remains unchanged throughout the entire control process as it is a parameter fixed friction model. Therefore, the accuracy requirements for the friction model are extremely high, but in actual systems, factors such as time and temperature affect friction, and fixed friction compensation terms inevitably have errors. For the method of fixed model friction compensation, it not only needs to meet the high requirements for model accuracy, but also needs to have better results in practical situations with extremely low environmental impact.

Han et al. [28] established a robust positioning control scheme using a friction parameter observer and a recursive fuzzy neural network based on sliding mode control. The friction state observer fully captures nonlinear dynamic friction, and uses recursive fuzzy neural network technology to establish an approximation method for system uncertainty, improving positioning accuracy. The performance of the control scheme is verified through experiments. However, for the purpose of more enhancement of precision positioning, the authors consider the robust control using the RFNN estimator and adaptive approximation error estimator.

Meng et al. [29] constructed an adaptive robust controller for dynamic friction compensation based on the LuGre friction compensation model. Adopting online recursive least squares estimation for model parameter identification, achieving real-time parameter updates, and designing a sliding mode controller to alleviate the degradation of control performance caused by parameter estimation errors and unmodeled dynamics. It is noted that since the trajectory is not always persistently exciting, the parameter estimates exhibit slow convergence. In Jin et al.'s work [30], a complementary sliding mode control method based on approximation angle saturation function is proposed, which

can reduce the boundary layer as the state trajectory changes until it converges to the sliding surface. This method effectively eliminates system chattering and improves position tracking accuracy and robustness. The designed control method was compared with the controller using traditional saturation function, and the results showed that the control method can achieve lower tracking error and stronger robustness. However, it can only be applied to the case where the trajectory of the state moves towards the switching surface, also the friction force is considered as a part of the lumped uncertainty, and the influence of friction force on the system is not properly analyzed.

The second type is error compensation based on specific friction models. This type of compensation method relies on establishing mathematical models that can accurately reflect various frictions in the system.

Lu et al. [31] proposed a sliding mode controller based on the approach law for controlling the position of permanent magnet synchronous motors. In addition, an adaptive disturbance observer was designed to suppress external disturbances and parameter uncertainties, and the designed controller was validated through experiments.

Chen et al. [32] proposed a discrete adaptive sliding mode controller for high-precision motion control of linear permanent magnet synchronous motors, which achieves real-time gain tuning capability and robustness, ensuring the performance of tracking tasks. Experimental results show that the designed controller has fast response and strong robustness to uncertainty and noise. However, maximum static friction force may arouse a higher tracking error at the beginning of the move.

Zhao et al. [33] proposed an adaptive neural network nonsingular fast terminal sliding mode control for high-precision position tracking of permanent magnet synchronous motors in the presence of uncertainty. Nonsingular fast terminal sliding mode control was used to achieve rapid convergence of the system at the equilibrium point. However, the approach often inadequately address parameter uncertainty, compromising the quality of identification outcomes

Currently, mainstream identification algorithms for LuGre friction model parameters include GAs, particle swarm optimization algorithms, and neural network algorithms. Despite the plethora of methodologies available for friction identification and compensation, challenges persist regarding parameter identification accuracy and chattering phenomena during feedforward compensation processes at velocity-zero positions [34-35].

To enhance speed and position tracking accuracy in high-precision, ultra-low-speed servo systems, an improved LuGre GA is employed for identification and compensation, achieving precise control objectives for the platform. Nonlinear friction links significantly impact the dynamic and static performance of high-precision, ultra-low-speed servo systems, largely evidenced by low-speed crawling, substantial static errors, or steady-state limit cycles. This work introduces the fundamental principles and mathematical expressions of the LuGre friction model and presents an advanced GA identification method based on LuGre. Simulation analyses demonstrate that, compared to traditional PID control, incorporating feedforward friction compensation based on the LuGre model effectively mitigates speed tracking discrepancies attributed to nonlinear friction, thereby enhancing both speed and position tracking accuracy while offering greater engineering practicality. In fact, these intelligent algorithms also have some shortcomings, such as poor generalization performance of NNA when processing large amounts of data, slow convergence speed of SAA, and low accuracy and easy divergence of PSO algorithm. Considering the multi parameter characteristics of the friction model established by the research object, this paper intends to use a genetic algorithm with relatively ideal parallel iteration ability as the identification method for the friction model.

Comment 9: The equations of LuGre coefficients in Eq.2 could have been introduced separately.

Answer:

We have introduced the equations of LuGre model individually, shown as

follows:

The model defines friction force $F_f(t)$ as a combination of bristle deflection dynamics and velocity-dependent terms.

1. Friction force:

$$F_f(t)=\sigma_0\cdot z(t)+\sigma_1\cdot\frac{dz(t)}{d(t)}+\sigma_2\cdot\dot{x}(t) \quad (2)$$

- σ_0: Bristle stiffness coefficient (N/m).
- σ_1: Bristle damping coefficient (N·s/m).
- σ_2: Viscous friction coefficient (N·s/m).
- $z(t)$: Average bristle deflection (m).
- $\dot{x}(t)$: Relative velocity (m/s).

2. Bristle Deflection Dynamics:

$$\frac{dz(t)}{d(t)}=\dot{x}(t)-\frac{\sigma_0}{g(\dot{x}(t))}z(t)\cdot|\dot{x}(t)| \quad (3)$$

The function $g(\dot{x}(t))$ models the Stribeck effect:

$$g(\dot{x}(t))=F_c+(F_s-F_c)\cdot e^{-(\dot{x}(t)/v_s)^2} \quad (4)$$

- F_c: Coulomb friction (N).
- F_s: Static friction (N).
- v_s: Stribeck velocity (m/s).

Comment 10: At section "Improved Annealing GA" the dictation of LuGre is incomplete.

Answer:

We thank the reviewer for pointing out this issue, we have added four more steps to the manuscript to complete the dictation of LuGre. We also redraw Figure 5 for better understanding. It is shown as follows:

The design of an enhanced genetic algorithm identification algorithm based on the LuGre model, with specific implementation steps as follows:

Step 1: Define the system parameters and population parameters for the friction model. Specify the population size: $\{x_k\}_{k=1}^{\omega}$ denotes both the upper and lower limits of the population, while the identification vector is represented as x_k.

Step 2: Implement adaptive calculations for the crossover rate and mutation rate. The crossover rate and mutation rate proposed in this study employ an adaptive strategy. The expressions for the crossover rate and mutation rate are as follows:

$$p_c = \begin{cases} p_{c\min} + \left(p_{c\min} - p_{c\min}\right) \cdot \left(f' / f_{avg}\right), & if \quad f' \geq f_{avg} \\ p_{c\max}, & if \quad f' < f_{avg} \end{cases} \tag{10}$$

$$p_m = \begin{cases} p_{m\min} + \left(p_{m\max} - p_{m\min}\right) \cdot \left(f' / f_{avg}\right), & if \quad f < f_{avg} \\ p_{m\max}, & if \quad f \geq f_{avg} \end{cases} \tag{11}$$

In Equations (10) to (11):

p_c denotes the crossover variation rate;

$p_{c\min}$ and $p_{c\max}$ represents the minimum and maximum values of the crossover rate, defined as constant parameters;

$p_{m\min}$ and $p_{m\max}$ indicates the minimum and maximum values of the mutation rate, which are also treated as constant parameters;

f refers to the higher fitness value of the two individuals involved in the crossover;

f_{avg} signifies the average fitness of each generation, as well as the average fitness value of the offspring.

Step 3: Implement crossover and mutation operations to generate the next generation population, During the parental crossover process, the participating parental individuals are denoted as $x_i^{l+1}(k)$ and $x_{i+1}^{l+1}(k)$. If the objective

function value of $x_i^{l+1}(k)$ exceeds that of $x_{i+1}^{l+1}(k)$, the resultant offspring individuals can be generated according to the following formula:

$$\left.\begin{aligned} x_i^{l+1}(k) &= \xi \cdot x_i(k) + (1-\xi) x_i(k+1) \\ \xi &= (\xi_{11}, \xi_{12}, \cdots, \xi_{1n}) \end{aligned}\right\} \tag{12}$$

In Equation (12): n represents the dimensionality of the variable, while parameter ξ can either be a constant or a random number that follows a uniform distribution.

$$\left.\begin{aligned} x_i^{l+1}(k) &= \eta \cdot x_i(k) + (1-\eta) x_i(k+1) \\ \eta &= (\eta_{11}, \eta_{12}, \cdots, \eta_{1n}) \end{aligned}\right\} \tag{13}$$

In Equation (13): Parameter η is defined as a constant or a random number $0 \le \eta \le 1$ that adheres to a uniform distribution. The mutation method employs single-point mutation. It is assumed that the mutation occurs in individual x_k, resulting in the new individual as follows [22]:

$$x_i(k) = x_0 \cdot \left(1 + \frac{\tan\left(rand(0,1) - 0.5\right)\pi}{2}\right) \qquad k = 1, 2, \cdots, N \tag{14}$$

Step 4: Simulated annealing for new species, initially, establish the number of optimal individuals as N_Best. The fitness experience of the first N_Best in each generation is unconditionally retained for the next generation, while each subsequent individual is saved with a certain probability following the Boltzmann acceptance criterion. The probability is given by:

$$R_i(x) = \begin{cases} 1 & f_1 \ge f_{N-Best} \\ e^{-(f_1 - f_{N-Best})/Te \cdot x} & f_1 < f_{N-Best} \end{cases} \tag{15}$$

In the equation (15):

Te represents the current temperature; f denotes the fitness of the current individual;

f_{N-Best} signifies the individual adaptation with the fitness ranking N_Best. Consequently, the individual fitness function can be chosen as:

$$\left\{\begin{matrix} G_{\max} = \max_{M}(J_m) \\ f_M = G_{\max} - J_{\max}\ or\ 1/J_m \end{matrix}\right\}(M = 1,2,\cdots,n) \tag{16}$$

Step 5: Assess whether the current iteration of the evolutionary process has concluded. If not, employ a ranking-based selection method to identify the population with the highest fitness value in the current generation, designating it as the new parent population. Return to Step 3. If the iteration is complete, proceed to Step 6.

Step 6: Extract and store the optimal solution derived from the single-round evolutionary process.

Step 7: Evaluate whether the predefined number of evolutionary rounds has been achieved. If not, revert to Step 2. If completed, advance to Step 8.

Step 8: Compute the fitness value of the optimal solution from each evolutionary round and select the global optimal solution for final output.

Step 9: Develop a genetic algorithm tailored to enhance the accuracy and performance of the LuGre friction model. The above steps can be represented by Figure 5.

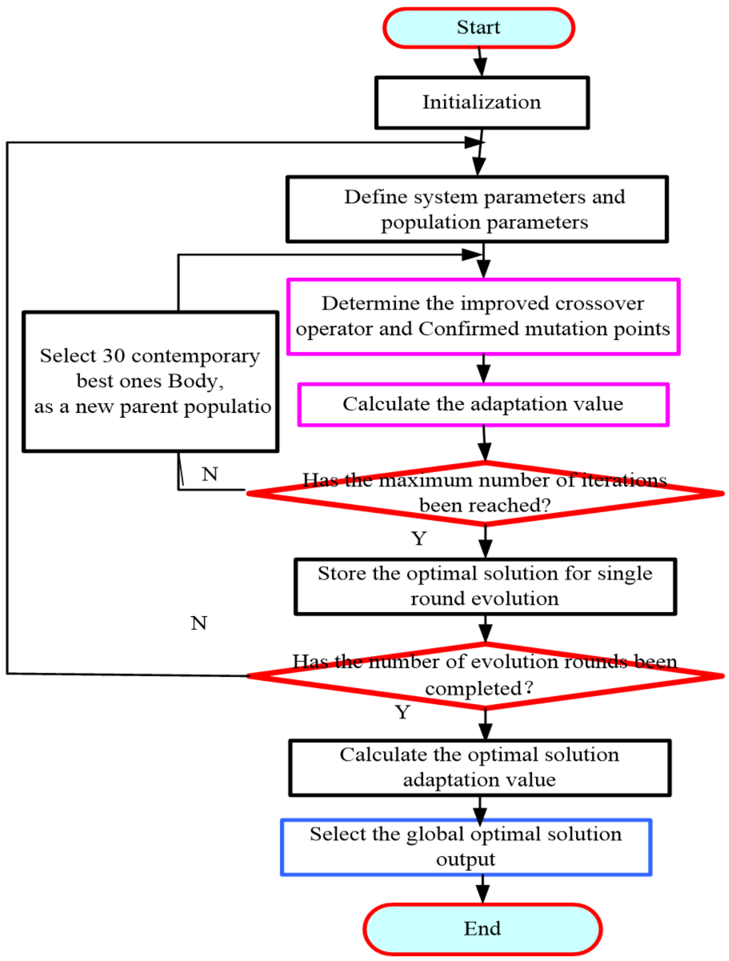

Fig. 5 Flowchart of Enhanced Genetic Algorithm Identification Algorithm Based on LuGre Model

Comment 11: The input of flowchart is incorrect at Fig.3.

Answer:

We thank the reviewer for pointing out this issue, we have changed Figure 3, shown as follows:

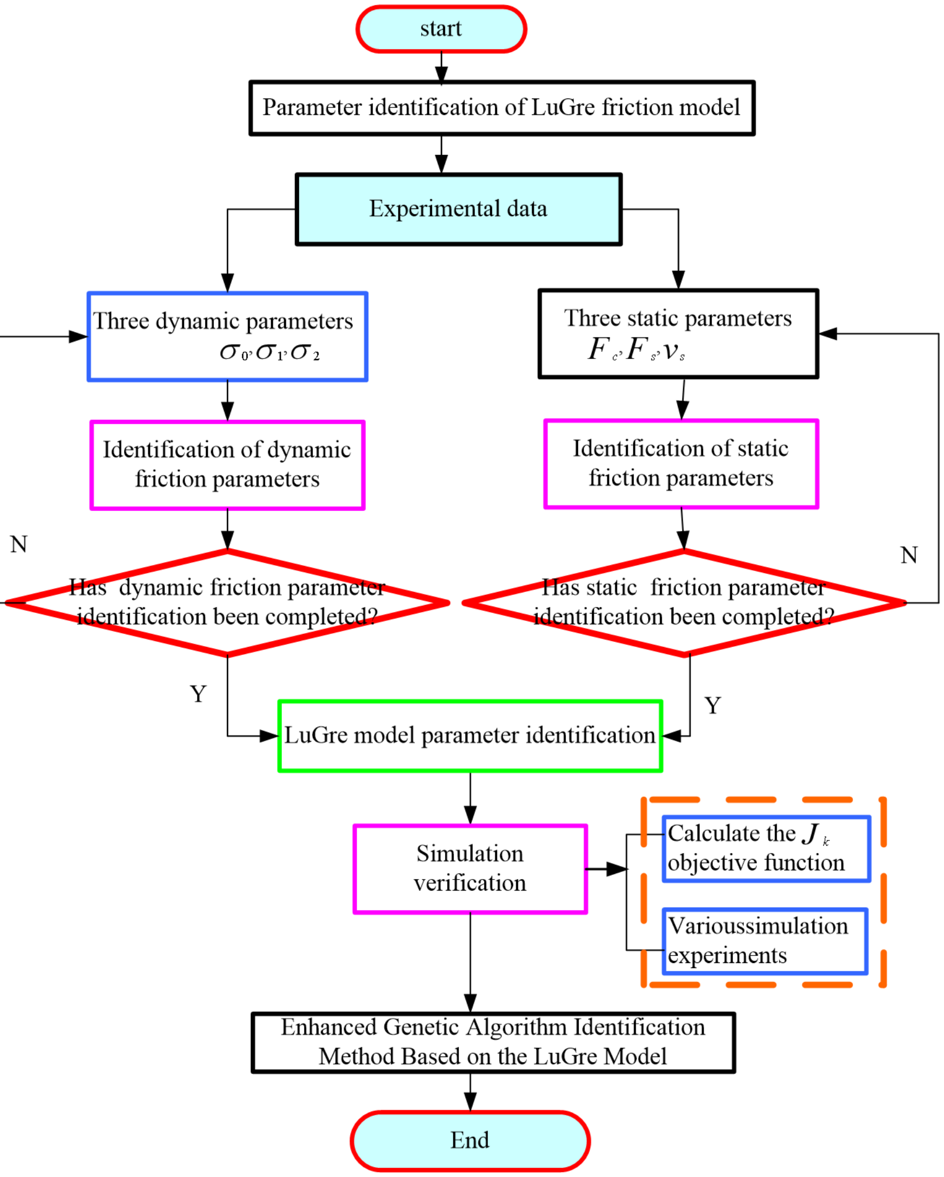

Fig.3 LuGre model parameter identification flow chart.

Comment 12: The error between two plots is considerable at Fig.11.

Answer:

We thank the reviewer for pointing out this issue, we checked Figure 11, and the figure is redrawn as follows:

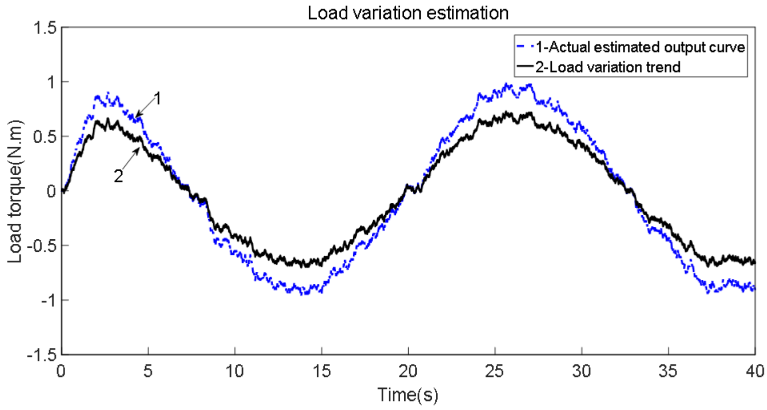

(a) Load variation estimation

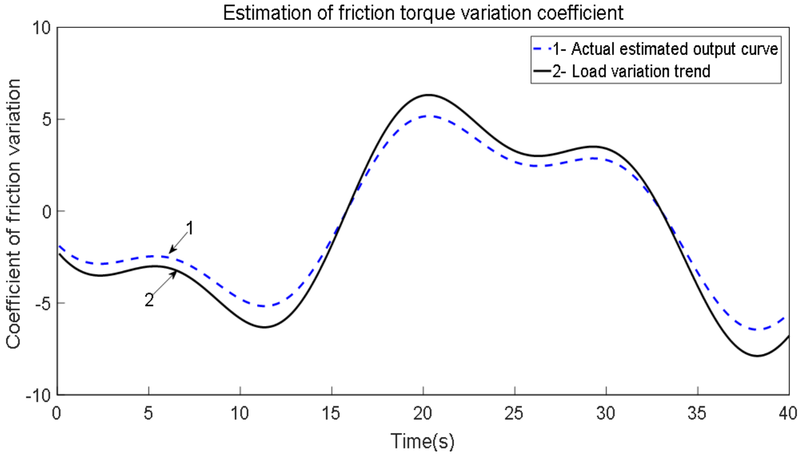

(b) Estimation of friction torque variation coefficient

Fig. 11 Estimation of load changes

Comment 13: What was the case of choosing a liner motor in order to evaluate a nonlinear friction model?

Answer:

The choice of a linear motor for evaluating a nonlinear friction model is driven by its ability to operate at ultra-low speeds, its sensitivity to friction effects, and its suitability for high-precision applications. These characteristics make linear motors an excellent platform for studying and validating advanced friction models like the LuGre model.

Nonlinear friction is a primary factor affecting the dynamic and static performance of servo systems driven by linear motors. The LuGre friction model has been widely adopted due to its accurate description of the dynamic and static characteristics of friction forces. The LuGre friction model of the

experimental system was identified through experimental and mathematical modeling methods. Based on this model, feedforward control technology was employed for friction compensation, and relevant experiments were conducted using the real-time TwinCAT3 software. The results demonstrate that friction compensation based on the LuGre model can eliminate the "dead zone" in velocity tracking and the "flat top" phenomenon in position tracking, thereby improving the accuracy of both velocity and position tracking in the system.

Comment 14: In Eq.1 the full formula has a parameter α as the power of e, why in this equation it was determined 2 and no other values?

Answer:

Currently, friction feedforward compensation in servo feed systems predominantly relies on the traditional Stribeck friction model, as illustrated in Fig. 1.

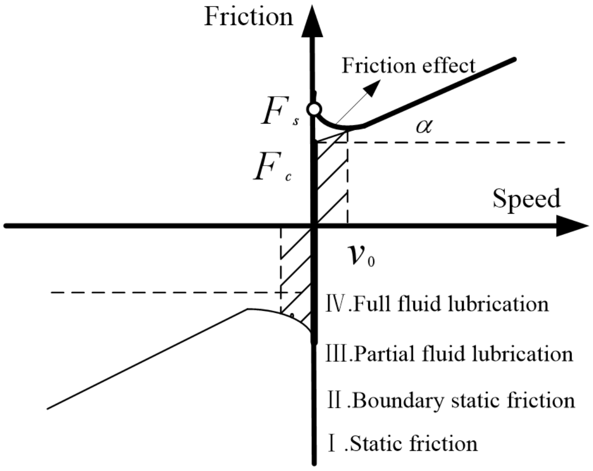

Fig.1 Traditional Stribeck friction model.

Traditional friction model expression:

$$F_f(v)=\left[F_c+(F_s-F_c)\cdot e^{-(v\cdot v_s)^{\delta}}\right]\cdot \mathrm{sgn}(v)+\alpha v \tag{1}$$

In Equation (1), $F_f(v)$ represents the total frictional force of the system; v represents the current motion speed of the system, $\mathrm{m/s}$; v_s denotes the Stribeck speed, $\mathrm{m/s}$; α is the coefficient of viscous friction, $\mathrm{N}s/m$; δ **is the**

Stribeck shape factor, generally, $\delta = 2$; F_c refers to the Coulomb friction force, N ; and F_s is defined as the maximum static friction force, N . Consequently, this defines the static coefficient.

Comment 15: On which contact surface do we have the deflection of bristles?

Answer:

We thank the reviewer for proposing such a good question, Canudas et al. proposed the LuGre model based on the Dahl model. This model posits that the contact between rigid body surfaces is mediated by elastic bristles, with the lower surface material exhibiting greater stiffness compared to the upper surface. When an external force is applied, the bristles undergo deformation due to the tangential force, generating frictional resistance. If the tangential force exceeds a critical threshold, the bristles experience further deformation, leading to sliding motion. A schematic representation of the surface contact in the LuGre model is illustrated in Figure 2.

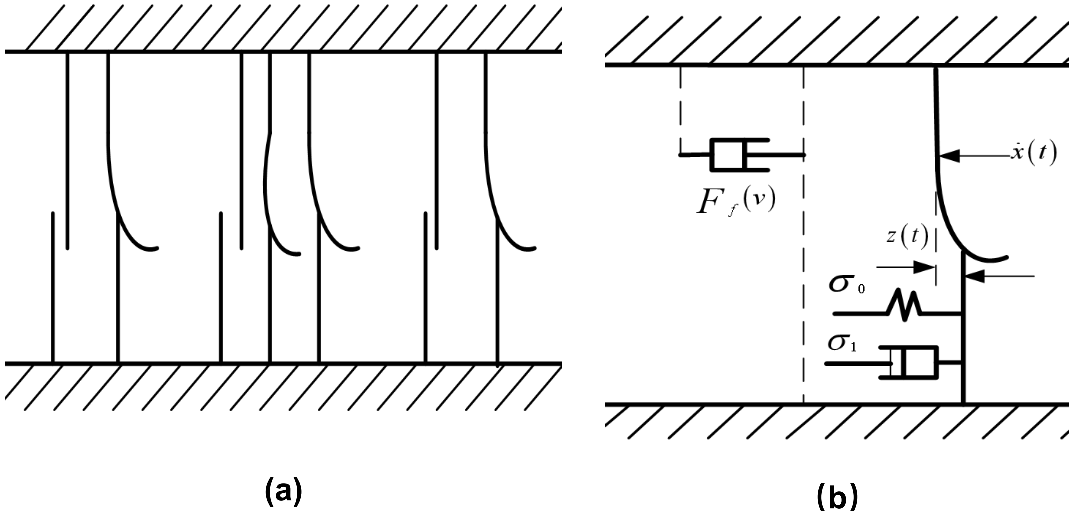

Fig.2 Lugre model object surface contact diagram: (a) Schematic diagram of bristle model distribution; (b) Motion detonation diagram of bristles

Comment 16: Why the identification matrix does not contain parameter σ_2?

Answer:

We are sorry for the missing of parameter σ_2, we have added it to the identification matrix $([\sigma_0 \ \ \sigma_1 \ \ \sigma_2 \ \ v_s \ \ F_s],t)$.

Comment 17: In Eq.6 the parameter "k" stands for number of irritations or time step since the parameter z(t) is a time dependent variable?

Answer:

In the LuGre friction model, z(t) is an unmeasurable internal state variable, and determining the model's parameters is also highly challenging. As a result, these parameters are typically obtained through offline parameter identification methods based on experimental data.

The enhanced genetic algorithm proposed in this study is specifically designed to address the issue of traditional genetic algorithms easily converging to local optima during the parameter identification of the LuGre friction model. Building on traditional genetic algorithms, this paper introduces an improved approach that mitigates the local optima problem in identifying parameters of the highly nonlinear differential LuGre friction model. This is achieved by initializing different parent populations for single-round evolution and performing multiple rounds of evolution, ultimately yielding the global optimal parameter solution. Prior to designing the improved genetic algorithm, the LuGre friction model described in Equation (5) was discretized to facilitate model implementation and program design. To enable the implementation of the model and program design, the LuGre friction model in Equation (6) is discretized as follows:

$$\begin{cases} z(k)=\left[\dot{x}(k-1)-\dfrac{\sigma_0}{g(k-1)}z(k-1)\cdot\left|\dot{x}(k-1)\right|\right]\cdot\Delta T+z(k-1) \\ g(k)=F_c+(F_s-F_c)\cdot e^{-(\dot{x}(k)/v_s)^2} \\ F_f(k)=\sigma_0\cdot z(k)+\sigma_1\cdot\dfrac{z(k)-z(k-1)}{\Delta T}+\sigma_2\cdot\dot{x}(k) \end{cases} \tag{6}$$

In Equation (6), k represents current sampling time. Initial condition: $z(0)=0$;

$\dot{z}(0)=0$ enter the corresponding speed $\dot{x}(k)$ to obtain LuGre friction.

Comment 18: The up to date references should be added to survey.

[1] P. Moradi, M. H. Korayem, N. Yousefi, "Extended Nonlinear Time-varying Lugre-based Friction Model Identification of Robot Manipulator with SMC Compensation Approach", Proceedings of the Institution of Mechanical Engineers, Part I: Journal of Systems and Control Engineering 237, no. 2: 207-219, 2023.

[2] P. Moradi, M. H. Korayem, N. Yousefi, "Online Identification and Robust Compensation of Extended Nonlinear Time-varying Friction Model in Robotic Arms", Journal of Mechanical Science and Technology 37, no. 1: 367-373, Jan 2023.

[3] M. H. Korayem, M. Zakeri, and M. Taheri, "Simulation of Two-Dimensional Nanomanipulation of Particles Based on the HK and LuGre Friction Models", Arabian Journal for Science and Eng., Vol. 38, No. 6, pp. 1573-1585, February 2013.

[4] M. H. Korayem, A. Hedayat, and S. F. Dehkordi, "Application of frictional contact to extend the functionality of cooperative manipulator chains in moving a common object in the form of closed kinematic chain", Applied Mathematical Modelling, Vol. 90, Pages 302-326, Feb. 2021.

Answer:

We thank the reviewer for helping improve the manuscript, we have added those up-to-date references to our manuscript.
